# Comprehensive Profiling of Zika Virus Risk with Natural and Artificial Mitigating Strategies, United States

**DOI:** 10.3201/eid2604.181739

**Published:** 2020-04

**Authors:** Michael J. Mina, L. Beryl Guterman, Kristen E. Allen, Saad B. Omer

**Affiliations:** Harvard T.H. Chan School of Public Health, Boston, Massachusetts, USA (M.J. Mina);; Harvard Medical School, Boston (M.J. Mina);; Jacobs School of Medicine and Biomedical Sciences, University at Buffalo, Buffalo, New York, USA (L.B. Guterman);; Emory University, Atlanta, Georgia, USA (L.B. Guterman, K.E. Allen);; Yale Institute for Global Health, New Haven, Connecticut, USA (S.B. Omer);; Yale School of Medicine, New Haven (S.B. Omer);; Yale School of Public Health, New Haven (S.B. Omer)

**Keywords:** Zika, transmission, control, viruses, United States, mosquitoborne diseases, vector-borne infections, viruses, risk profiling

## Abstract

Zika virus is transitioning to become a long-term public health challenge, and countries should remain informed of the risk for emergence. We developed a stochastic epidemiologic model to profile risk for Zika virus emergence, including trimester-specific fetal risk across time, in all 3,208 counties in the United States, including Puerto Rico. Validation against known transmission in North America demonstrated accuracy to predict epidemic dynamics and absolute case counts across scales (R^2^ = 0.98). We found that, although sporadic single transmission events could occur in most US counties, outbreaks will likely be restricted to the Gulf Coast region and to late spring through autumn. Seasonal fluctuations in birth rates will confer natural population-level protection against early-trimester infections. Overall, outbreak control will be more effective and efficient than prevention, and vaccination will be most effective at >70% coverage. Our county-level risk profiles should serve as a critical resource for resource allocation.

Zika virus is a flavivirus spread by *Aedes* mosquitoes that for >60 years remained only an esoteric threat to human health (*1*). However, the recent Zika epidemic, which erupted in South America in 2015 and became the largest in history, brought the virus to prominence, particularly because infection has been linked to fetal microcephaly and other neurodevelopmental and neurologic sequelae (*2*).

Although no longer classified a global emergency by the World Health Organization (WHO), Zika virus emergence and transmission continues globally, and WHO warns that Zika virus is set to remain as a long-term public health challenge (*3*). Given the critical importance of preventing Zika virus infections, especially during pregnancy, transmission anywhere requires that nations remain vigilant and informed at local, state, and national levels to prevent and control introduction and onward transmission (*4*,*5*). This imperative is especially important for countries such as the United States that simultaneously harbor the *Aedes* vectors and maintain essentially entirely susceptible populations.

Numerous models for the potential emergence of Zika virus in the United States focus largely on the ecologic niche of *Aedes* mosquitoes (*6*–*10*). Projections that simultaneously consider vector dynamics and human demographics, including birth seasonality, to resolve both relative and absolute epidemic risk and potential control measures across space and time throughout the year are more limited.

Here we present a stochastic Zika virus compartment model that considers the overlap of vector dynamics and human demographics at the county level in the United States, including Puerto Rico. The model was used to profile the risk for Zika virus transmission, assuming an initial introduction into each county, including trimester-specific fetal exposures for each of the 3,208 counties and municipalities within the United States including Puerto Rico over time and under varying control measures. We tested 3 approaches to controlling Zika virus transmission and assessed their utility in preventing or abrogating Zika virus transmission. These approaches include reducing human–vector contact (i.e., behavior modification and ubiquitous technologies such as air conditioning, screens, and long clothing); depleting adult vectors (i.e., mosquito fumigation programs); and vaccination, which, should a successful candidate vaccine come to market, might reduce individual and community risk for infection once herd-immunity thresholds are achieved (*11*).

## Methods

We modeled county-level Zika virus transmission using a coupled 2-system stochastic human–mosquito differential equation compartment model (Appendix Figure 1). The human system was a susceptible-exposed-infected-recovered model and the mosquito system a susceptible-exposed-infected compartment model that incorporates vector and viral life-stage dynamics as functions of temperature throughout the year, as well as climate (temperature) and demographic data, including county- and municipality-level seasonality of births. More specifically, we coupled high-resolution *Aedes* vector risk maps (*12*) describing the ecologic extent of the major vectors of Zika virus, *Ae. aegypti* and *Ae. albopictus* mosquitoes, with dynamic temperature-dependent Zika virus and *Aedes* life-stage models, local climate data, and county-level demographic information, including population and monthly birth cohort data. We simulated Zika virus transmission given a single importation (index case) into each county across time and under varying control measures. We simulated stochastic trajectories by using an adaptation of the fundamental Gillespie stochastic simulation algorithm, an adaptive tau leaping procedure (*13*) for continuous-time Markov processes, which we implemented by using the AdaptiveTau R package (*13*).

For each county and each scenario, we conducted >500 simulations and derived probability of initiation of a transmission chain from the index case, expected outbreak size when transmission occurs, and, by fitting nonlinear models to county-level monthly birth data, trimester-specific fetal Zika virus exposures. To remain relevant to local, state, and national entities, all 3,208 counties and municipalities were investigated independently, assuming only that an index case-patient arrives in the county, regardless of origin (i.e., spread from a neighboring county or an international import).

### Model Parameters

We selected parameters from ecologic and epidemiologic literature (Appendix Table 1; Appendix Figure 1). Given the novelty of the Zika virus as a major human pathogen, relatively limited information on its dynamic life-stage properties is available. Thus, properties relating to transmission and extrinsic incubation period were borrowed from the large body of literature on dengue virus dynamics, because dengue virus is a closely related but more completely studied mosquitoborne flavivirus that shares the same mosquito vector host system as Zika virus. Such a strategy is commonly used in modeling for emerging pathogens, including other Zika virus transmission models (*7*,*14*).

### Trimester-Specific Pregnancies and Exposure Calculations

Infection with Zika virus is most concerning during pregnancy, where maternal infection has been linked to congenital birth defects, most notably microcephaly (*15*). These defects appear to be most strongly associated with Zika virus infection during the first and second trimesters (*16*,*17*). Therefore, we derived trimester-specific maternal–fetal exposures from county-level demographic data, including birth seasonality.

Throughout the year, the proportion of a population’s births fluctuate in a predictable manner across calendar months. To estimate the numbers of children born per month, and thus calculate expected numbers of first-, second-, and third-trimester pregnancies per month, we used the number of births per month for each county over an 8-year period (2007–2014) based on US Census data. For each county, we fit generalized additive models to the monthly data to estimate the fraction of annual births per month for each county. We then coupled the county-level generalized additive model output, indicating the expected proportion of annual births in each calendar month, to annual birth numbers for each individual county to calculate monthly county-specific expected pregnancies.

From the monthly birth data for each county, we back-calculated the time of conception, assuming a 40-week gestation and a constant rate within a given month. On the basis of this calculation, we derived pregnancy cohorts, defined as the number of women becoming pregnant per month of the simulation, which allowed us to follow each cohort throughout their pregnancies and evaluate the number of pregnancies in their first, second, or third trimester during each month for each county or municipality. To calculate infections during pregnancy during the simulations, we derived the number of fetal exposures per week per trimester by drawing simultaneously from 3 binomial distributions each week, each with the size equal to the number of first-, second-, or third-trimester pregnancies in the county during the week of interest, and with a probability equal to the proportion of the population infected during that week (Appendix). By drawing from a binomial distribution, we incorporated stochastic effects that influence the number of infections among gravid women, relative to the proportion infected across the population as a whole.

### Model Validation

We compared models by comparing reported or published estimated incidence and case counts for known Zika that have arisen from local transmission in the United States including Puerto Rico against the respective simulated data. In addition, given similarities between Zika virus and dengue virus, such as common transmission vectors and dynamics (*18*), we compared reported or published estimated incidence and case counts for known dengue outbreaks that have arisen from local transmission in the contiguous United States against the respective simulated data. Validation data for Brownsville County, Texas, and Miami-Dade County, Florida, came from the Texas and Florida departments of health, respectively (*19*,*20*). Validation data for Monroe and Martin counties in Florida came from serosurvey data collected by the Florida department of health and the Centers for Disease Control and Prevention (CDC) (*21*,*22*).

Model validation for Puerto Rico used 2 resources to derive estimates of monthly and cumulative Zika virus incidence across Puerto Rico’s 8 health regions. Official reported data from the Puerto Rico Ministry of Health (http://www.salud.gov.pr/Estadisticas-Registros-y-Publicaciones/Pages/VigilanciadeZika) provided information on the monthly dynamics (fractional abundance) of the epidemic for each health region, namely the proportion of cases per month in each of the 8 health regions. Separately, to obtain best estimates for total cumulative incidence, we used a recent and thorough report from CDC by Chevalier et al. (*23*), which analyzed blood donor screening data from April 3 through August 12, 2016, from the 2 largest blood banks in Puerto Rico to estimate overall epidemic size. The use of both resources used the strengths of both types of reports (accurate fractional abundance over time and in each health region, and accurate estimates of cumulative incidence) to derive best estimates for model validation (Appendix).

## Results

**Figure 1:**
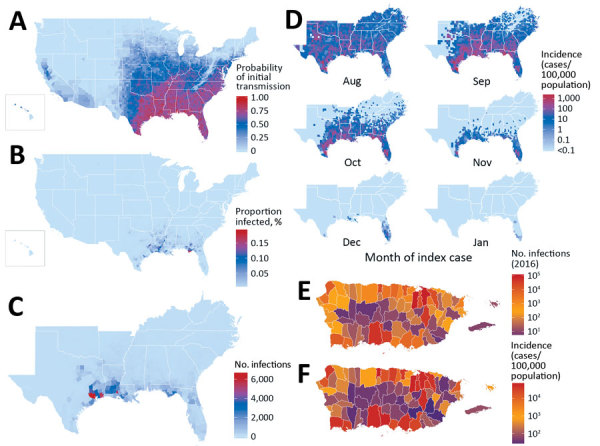
County-level Zika virus risk profiling, United States including Puerto Rico. A) Probability of initial transmission from an index case introduced during peak vector abundance, calculated as the proportion of simulations with >1 transmission event, for every county. B) Proportion of population infected. C) Total case counts for the southeastern United States (nationwide data depicted in Appendix Figure 6) when transmission does occur after index cases during peak abundance (median calculated among simulation with >1 transmission event). D) Monthly incidence and duration of outbreaks. Shown is the median monthly incidence of Zika virus infections from August index cases. E) Total number of simulated exposures in Puerto Rico ending December 31, 2016. F) Final epidemic size (incidence) at the end of simulations. For panels E and F, imports into each municipality corresponded temporally with initial cases reported in 2015 and 2016. All simulations assess counties and municipalities independently.

Across the United States, when an infectious Zika virus–infected person was introduced during peak *Aedes* abundance for each county (Appendix Figure 2), the model predicted at least minimal transmission (defined as >1 transmission event in >0.05% of simulations) in 86% of US counties (Figure 1, panel A), essentially reflecting the limit of *Aedes* mosquito distribution (Appendix Figure 3). However, the probability of any transmission varied widely and was focused in the Southeast United States, Puerto Rico, and portions of Texas (Figure 1, panel A; Appendix Figures 4–6).

Once initiated, transmission chains were very limited. Of counties where the model indicated at least minimal transmission from index case-patients during peak vector abundance, 93% of transmission chains (interquartile range [IQR] 88%–98%) had median incidence (among simulations with transmission) of <1% of the population (Figure 1, panel B), and 63% (IQR 48%–78%) of chains had final outbreak sizes of <10 total cases (Figure 1, panel C; Appendix Figure 6). Where *Ae. aegypti* mosquitoes are scarce compared with *Ae. albopictus* mosquitoes (Appendix Figure 7, panel A), 95% of counties had median outbreaks of <10 total cases (Appendix Figure 7, panel B), demonstrating that onward transmission is driven primarily by *Ae. aegypti* mosquitoes.

Along the Gulf Coast, outbreaks were more sustained. In Harris County, Texas, home to the Houston metropolitan area (population ≈4.8 million), the model predicted the largest epidemics in the 50 states, with a median epidemic size of 6,538 infections (IQR 1,846–17,440 infections) from an import during peak vector abundance. Although the entire Gulf Coast region is at risk for outbreaks, only 3 states contributed 97% of the top 100 counties with the largest simulated outbreaks: Florida (40%), Texas (35%), and Louisiana (22%). Mississippi contributed the other 3%.

According to our model, no counties within the 50 states sustained transmission beyond the first winter (Figure 1, panel D; Appendix Figure 8), although Miami-Dade and Broward counties in Florida sustained transmission as late as February in a fraction of simulations. Only Puerto Rico, Hawaii, and select counties, primarily in Florida and Texas, supported any transmission from the index cases occurring outside of the late spring through early autumn months (Appendix Figure 9, 10). Within the 50 states, only Miami-Dade County had evidence of transmission as early as February, and outbreaks there were limited in size (median 2 cases).

Our model showed final epidemic size was particularly sensitive to time of introduction (Appendix Figure 11), especially among counties most susceptible to transmission. Among the top 10% of counties by predicted final epidemic size, the time of import that maximized incidence was ≈2 months earlier than that which maximized initial transmission (May vs. July; p<0.001) (Appendix Figure 12, panels A, C), and final incidence was as much as 10-fold greater. This difference disappeared among the 80% of counties with the smallest predicted final epidemic size (Appendix Figure 12, panels B, D), where both metrics were maximized by imports during peak vector abundance, reflecting very limited transmission chains in most counties.

In Puerto Rico, simulated epidemics were more sustained and greater in magnitude. When index case-patients were introduced into each municipality to match timing of first reported cases in the 2016 epidemic (*6*), through 2016 our model detected 479,025 (IQR 310,365–662,257) total infections (Figure 1, panel E), representing a median incidence of 14% (IQR 9%–19%) of the population. The model also showed that San Juan (population 365,576) had the largest epidemics, which usually persisted for up to 3 years and infected 58% (IQR 52%– 74%) of the population (Figure 1, panel F; Appendix Figure 13). These findings are consistent with previous Zika epidemics among island populations, where seropositivity reached 50%–70% (*24*,*25*). Across simulations, the total incidence on the island of Puerto Rico was 24% (IQR 19%–30%), suggesting that most infections had already occurred in 2016, when the index case was introduced. In addition, only 19% (IQR 13%–23%) of the municipalities in the model sustained transmission through the first winter, whereas 14% and 3% sustained through the second and third winters, respectively.

### Zika Virus Infections during Pregnancy

**Figure 2:**
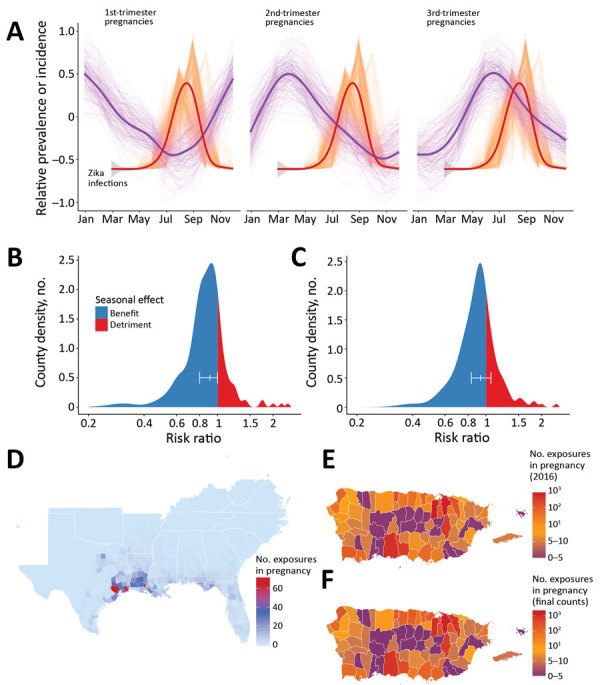
Zika virus infections during pregnancy and effects of natural birth dynamics, United States including Puerto Rico. A) Standardized prevalence of first-, second-, and third-trimester pregnancies throughout a year in the southeastern United States and Texas are plotted against the simulated and standardized Zika epidemic curves for each county and for every month of import. Thin purple lines indicate county-specific prevalence of pregnancy in each respective trimester, and thick purple lines show a generalized additive model fit. Thin orange lines indicate median outbreak per county, including distinct lines for each month of import during March–November. Thick orange line is a generalized additive model fit to the county-level data. B, C) Zika virus exposure risk ratio and 95% CI during (B) first (versus third) and (C) second (versus third) trimester of pregnancy, driven by the dynamics depicted in panel A (Appendix Table 2). D) Median number of infections (simulated) during pregnancy when index cases are imported during peak vector abundance. E, F) Median number of infections (simulated) during pregnancy for each municipality (E) in Puerto Rico in 2016 and (F) over the entire epidemic. Data in panels E and F include index cases that were introduced into each municipality to correspond with initial introductions reported in the current epidemic

We found that natural seasonality in births results in waves of first- and second-trimester pregnancies that are out-of-phase with peak Zika virus infections in the model (Figure 2, panel A) and thus confers significant population-level protection against early-trimester exposures (Figures 2, panels B, C). Our model indicates that birth seasonality alone reduced risk for Zika virus exposure during first- (versus second- and third-) trimester exposures by 11% (relative risk 0.89 [95% CI 0.80–0.99]; p = 0.012).

Although counties in our model with the greatest numbers of fetal exposures generally tracked with epidemic size, distinct demographics led to deviations. In particular, Florida contributed 12% fewer counties to the top 100 counties, when ordered by rates of fetal exposures versus rates of total infections. Within the continental United States, Harris County, Texas, had the highest number of exposures during pregnancy (78 [IQR 20–183] exposures) after introduction of index cases during peak vector abundance (Figure 2, panel D; Appendix Figure 14). In Miami-Dade County, when simulated with a July index case introduction to match the 2016 outbreak (see also model validation below), we detected only 1 (IQR 0–3) fetal exposure from locally transmitted infections.

In Puerto Rico, when index cases were introduced into each municipality to match timing of initial cases reported in the current outbreak, through 2016 we detected 4,187 [IQR 2,733–5,760] infections during pregnancy (Figure 2, panel E), representing 10% (IQR 6%–13%) of all births. Throughout the entire simulated epidemic in Puerto Rico, the IQR for exposures in pregnancy was 5,800–9,100 (Figure 2, panel F).

### Control Strategies

**Figure 3:**
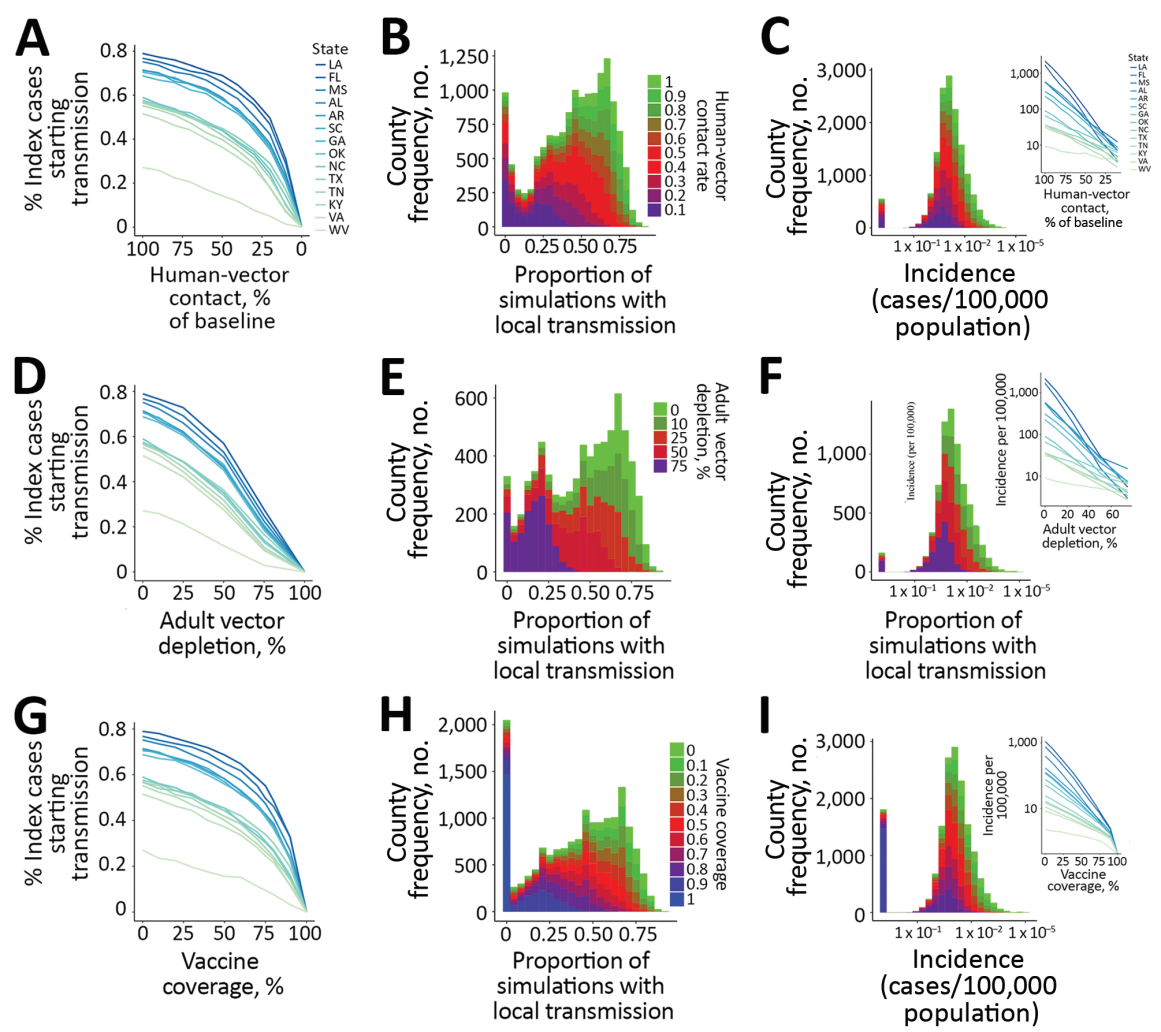
Zika prevention and control strategies, United States. For each county in the United States including Puerto Rico, classes of prevention or control strategies were assessed, including (A–C) reductions in human–vector contact, (D–F) adult vector depletion, and (G–I) vaccination. A, D, G) Proportion of index cases initiating >1 transmission event versus extent of each intervention. Each line represents the statewide average across each of the constituent county’s median simulations. B, E, H) Histograms depicting number of counties versus probability of permitting >1 transmission event from the index case, color coded by the level of each respective intervention. C, F, I) Histogram showing the number of counties versus incidence across levels of respective intervention (color coding as in panels B, E, and H). Insets in panels C, F, and I show incidence (on a log-linear scale) versus extent of each respective interventions.

When human–vector contact rates were reduced from baseline in the model, the probability of initial transmission remained relatively insensitive, until contact was reduced by >70%, at which point initial transmission fell sharply (Figure 3, panels A, B). Incidence was exquisitely sensitive to reductions in contact, and fell log-linearly, with the magnitude of the slope proportional to the baseline incidence (Figure 3, panel C).

Depletion of adult *Aedes* mosquitoes through reductions in *Aedes* mosquito average lifespan in the model was effective at decreasing likelihood of initial transmission and epidemic size across all levels of intervention, again with incidence more sensitive than initial transmission from the index case (Figure 3, panels D–F).

Vaccination was relatively more effective at preventing initiation of transmission than reducing incidence, particularly once vaccination coverage exceeded 70% (Figure 3, panels G–I). This finding is consistent with an R_0_ for Zika virus of 3–4, based on the simple but robust formula for the vaccination coverage *V*) required to achieve herd immunity: *V* = 1–1 / R_0,_ where R_0_ is the basic reproductive number), in agreement with previous estimates (*26*,*27*).

### Model Validation

**Figure 4:**
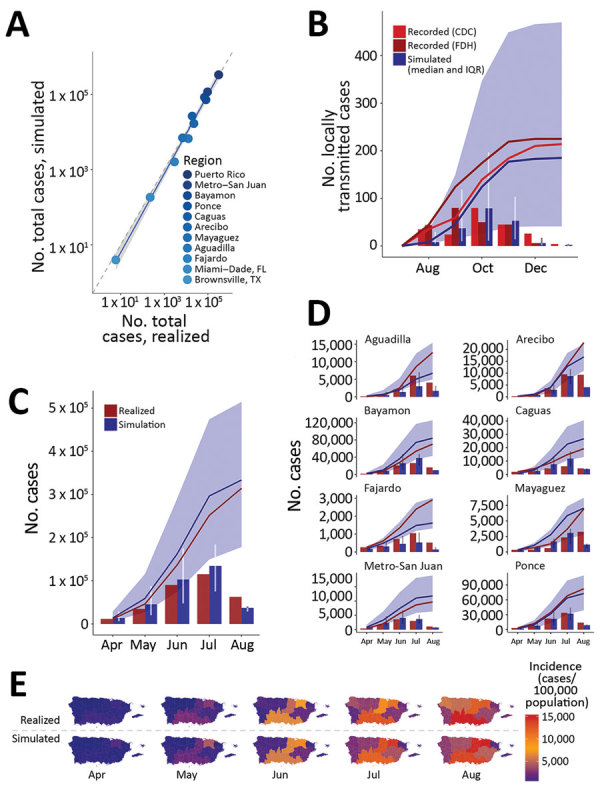
Model validation for Zika virus infection cases reported or estimated and simulated for outbreaks, United States including Puerto Rico, 2016. A) Total cases (median simulated) versus total cases reported or estimated (realized) for each of the regions are plotted as a scatter plot. Dotted line indicates 1:1 relationship. B–D) Monthly and cumulative simulated cases are plotted against reported or estimated cases for (B) Miami–Dade County, Florida, (C) Puerto Rico, and (D) each of the 8 health regions of Puerto Rico. Dark blue columns and line in panel B show monthly and cumulative case counts for the median simulated outbreak (among simulations with >1 transmission event) and shaded region shows the interquartile range. Red columns and red solid lines indicate the respective monthly and cumulative cases recorded or estimated, as noted. Data in panels C and D are as in B, but summed over the constituent municipalities (i.e., the dark blue line in panel C shows the sum of the cumulative case counts for each municipality in Puerto Rico). For panels C and D, validation data were available only for April 3–August 12, and thus realized and simulated case counts represent only cases measured or predicted within this period. E) Cumulative cases realized (upper panel) and simulated (lower panel) for each health region of Puerto Rico during April–August 2016. Validation data were available only for April 3–August 12, and thus realized and simulated case counts represent only cases measured or predicted within this period. CDC, Centers for Disease Control and Prevention; FDH, Florida Department of Health; IQR, interquartile range.

We validated the model against known Zika outbreaks in the United States including Puerto Rico since late 2015, including Miami-Dade County (Florida), Brownsville County (Texas), and Puerto Rico, with separate tests across each of Puerto Rico’s 8 health regions. Overall, the model accurately predicted the dynamics and absolute case counts for each site tested (R^2^ = 0.980; p<0.001; Figure 4, panel A). In Miami-Dade County, where Zika virus transmitted locally in 2016, the model estimated a total epidemic size of 185 (IQR 45–467) cases (Figure 4, panel B), in strong agreement with the 225 locally transmitted cases reported by the Florida Department of Health and the 214 reported by CDC (*20*,*28*,*29*). In Brownsville County, Texas, where local Zika virus transmission was detected during October–December 2016 and infected 6 persons (*19*), the model estimated a median of 4 cases (IQR 1–8).

Because much of the model is parameterized on the basis of existing biological data measured for dengue viruses, we also validated the model against known dengue outbreaks in Florida. In Monroe County, Florida, local dengue transmission was detected in September 2009. A serosurvey conducted in the surrounding areas of the locally acquired cases estimated an infection rate of 3%–5% among residents during July–September 2009, where 5% includes presumptive infections in addition to acute and recent infections. The model estimated a median proportion infected of 1.4% (IQR 0.07%–3.38%) (*21*,*22*). In Martin County, Florida, local dengue transmission was detected in August 2013 and resulted in 22 cases. By late September 2013, a serosurvey in the surrounding area of the reported cases estimated a total of 29 cases. Given an import in early August, the model estimates a median of 69 (IQR 11–236) cases; for September import, the estimate is 14 (IQR 3–33) cases (*22*,*30*). When index cases were introduced into each municipality in Puerto Rico to correspond temporally with initial cases per health region reported by the Puerto Rico Ministry of Health (*6*), the model accurately predicted the monthly and cumulative case counts in Puerto Rico (325,000 infections vs. 314,209 simulated infections; Figure 4, panel C) and performed nearly as well for each of Puerto Rico’s 8 health regions (Figure 4, panels D, E; Appendix Figure 15), each representing an independent validation set. Across the 10 independent sites that we were able to validate the model against, the actual (realized) incidence was within the IQR of our simulations, and usually within a single-fold difference from the median simulation.

## Discussion

Overall, our model predicts interventions would be more effective at preventing additional transmission initiated by the index case than reducing the probability of an outbreak taking place. Therefore, given limited resources, a reactive approach focused on infection control rather than complete prevention might prove most beneficial. However, for those counties with highest overall invasion potential (Appendix Figure 16), early strategies aimed at preventing any transmission might be warranted, especially areas with high rates of potential imported cases (e.g., southeastern US cities with international airports). A vaccination coverage >70% would be most effective in preventing future outbreaks in these high-risk areas. However, pockets of unvaccinated persons are associated with elevated risk for infectious disease outbreaks (*31*). Therefore, this threshold might vary given a nonhomogeneous spatial distribution of vaccination coverage. In addition, these counties might serve as optimal US settings for Zika vaccine efficacy trials.

Zika outbreaks are likely to be highly restricted by both time and space, limited within the 50 US states almost exclusively to the summer months and the Gulf Coast region, where the *Ae. aegypti* mosquito vector is most abundant. Although our model predicts many counties within the United States could support >1 transmission event from an index case, nearly all transmission outside of this region was extremely limited. Therefore, outside of the southeastern United States, a detected transmission event from an imported case will most likely represent only a sporadic occurrence, with minimal onward transmission even in the absence of local control efforts. These transmission patterns are consistent with previous local transmission patterns of other mosquitoborne flaviviruses in the United States, such as chikungunya and dengue, and with other model estimates (*9*,*32*).

Our model also shows that few municipalities in Puerto Rico sustained transmission through the first winter, with even less transmission sustained through the second and third winters. This finding suggests that sustained transmission throughout Puerto Rico requires continued case exports from municipalities with uninterrupted transmission. These findings are also consistent with CDC’s reported Zika virus disease trends among travelers for 2016 and 2017, which show a decrease in the number of reported cases, from 4,205 cases in 2016 to only 331 cases in 2017 (*32*).

We also found that the natural seasonality in human births will likely serve to reduce population risk for early-trimester infections, which alone should serve to abrogate the number of fetal exposures resulting in neurologic complications. In addition, planned seasonal conception (based on birth seasonality and local Zika virus transmission data) is a viable intervention to pursue while maternal Zika virus vaccine and risk profiles, as they relate to gestational age, are being developed (*33*,*34*). Our model showed that in Puerto Rico, most fetal exposures occurred within the first year of the epidemic, suggesting that most fetal exposures have already occurred in 2016. Previous estimates by Ellington et al. (*29*) using Zika case data projected that ≈7,800 exposures in pregnancy would occur through 2016. Of note, that study anticipated an overall population incidence of 25% through 2016, whereas more recent estimates (*23*) place the actual incidence closer to 15%–20%. Thus, Ellington et al. might have overestimated actual fetal exposures by 20%–40%, which, when corrected for the updated incidence, places our model estimate well in line with theirs.

Some limitations to the proposed model might influence county level risk profiles for Zika virus transmission. For areas with a high level of travel-associated imports of Zika virus cases, such as cities with cruise ship ports and large airports, the associated risk might be an underestimate because our model does not consider multiple imports of infectious persons (*35*). In addition, certain parameters (e.g., incubation period and period of infectiousness of Zika virus infection) and transmission pathways (e.g., sexual transmission of Zika virus) are not fully understood and might contribute to elevated risk for Zika epidemics (*36*,*37*).

Zika virus transmission is expected to persist as a long-term public health challenge, and the United States remains an entirely susceptible population, with risk for transmission. As long as Zika virus circulates anywhere, the continued importation into the United States remains a potential risk. Our comprehensive profiling efforts should serve a critical need for decision making across all levels of government regarding efficient use of local, state, and national resources aimed at preventing and controlling Zika virus transmission and should provide critical information to inform future vaccination efforts.

Appendix Additional information about comprehensive profiling of Zika virus risk with natural and artificial mitigating strategies, United States.
