# Supplementary material for: Comprehensive Profiling of Zika Virus Risk with Natural and Artificial Mitigating Strategies, United States
Source: Emerg Infect Dis. 2020 Apr;26(4):700–10. doi: 10.3201/eid2604.181739 (PMC7101119; doi:10.3201/eid2604.181739)
Supplement: Appendix — Additional information about comprehensive profiling of Zika virus risk with natural and artificial mitigating strategies, United States. [file 18-1739-Techapp-s1.pdf]

Article DOI: https://doi.org/10.3201/eid2604.181739

Comprehensive Profiling of Zika Virus Risk with Natural and Artificial Mitigating Strategies, United States

Appendix

Materials and Methods

Model Overview

The SEIR-SEI human-vector model (Appendix Figure 1) is based on the Ross-Macdonald set of simplifying assumptions, namely that following a latent period, a pathogen is passed from an infected mosquito to a susceptible vertebrate host and from an infected vertebrate host to a susceptible mosquito during blood feeding (*1*,*2*). Once recovered from infection, immunity is considered absolute and not waning. Zika virus transmission between human and vector populations is dependent on: the number of mosquitos per human per day, the number of blood meals on humans per mosquito per day and the probability of transmission from an infectious mosquito to a susceptible human, and vice versa. Our model expands upon the basic Ross Macdonald model by integrating both *Aedes aegypti* and *Aedes albopictus* vectors, and their separate temperature dependent life-stage dynamics. Of note, by explicitly modeling vector life-stage and feeding as functions of temperature, the mosquito-human interaction rates change dynamically over the course of simulations (e.g., the number of mosquitos per human will differ markedly between summer and winter months). As well the extrinsic incubation period (EIP) is temperature dependent and modeled as a temperature dependent function. Thus our model incorporates a dynamic description of virus-vector-host interactions, parameterized from the best available information in the literature, described below, enabling simulations to extend over multiple years, while avoiding common limiting assumptions such as constant human:mosquito rates across time throughout the year. On the other hand, given the novelty of the Zika virus as a major human pathogen, there is relatively limited information on its dynamic life-stage properties. Thus, a limitation of the current study is that numerous properties relating to

transmission and EIP are borrowed from the large body of literature surrounding dengue virus dynamics, as it is a closely related but more completely studied mosquito-borne flavivirus that shares the same mosquito vector host system. Such a strategy for closely related viruses is common for emerging pathogens, and has been successfully employed for other Zika virus transmission models (*3*,*4*).

Spatial Properties

To best inform at the county-level (and by extension also inform at state and national levels), our aim was to describe the potential for Zika virus transmission within each county and municipality independently. Simulations thus assume that an index case arrives in that county, regardless of whether the case arrives from a neighboring county or via a traveler from a distant country. The current investigation therefore provides information on potential for Zika virus transmission in a given county in a manner that is source agnostic, and thus equally useful for a county sharing a border with a nearby county with an ongoing epidemic, or a county likely to receive travelers coming from Zika infected regions.

Model Parameters

Mosquito-Human Interactions per Day (m)

For each county, a maximum number of mosquitos-human interactions per day ($\lambda = m_{max}$) during peak vector abundance was calculated using high resolution (5km x 5km) maps detailing the global spatial distribution of *Aedes aegypti* and *Aedes albopictus* (*5*). In brief, these maps, produced by Kraemer et al. were developed by coupling the largest contemporary database of known geographic occurrence of both *Aedes aegypti* and *Aedes albopictus* (n = 19,930 individual observations) with pertinent environmental layers (temperature, precipitation, vegetation and urbanization) to derive what are widely considered the best available data on the spatial distributions of *Aedes aegypti* and *Aedes albopictus* occurrence probabilities. Additionally, these global maps, which are freely available for download (http://goo.gl/Zl2P7J) agree with the estimated range of *Aedes aegypti* and *Aedes albopictus* in the U.S. as put forth by the U.S. Centers for Disease Control and Prevention (*6*,*7*). Using these high resolution *Aedes* occurrence probability maps, we calculated mean probability of occurrence of *Ae. aegypti* and *Ae. albopictus* for each county or municipality using border data retrieved from the Global Administrative Areas or GADM database, freely available at http://www.gadm.org, and easily

accessible in R using the getData() function of the Raster package (*8*). The mean occurrence probability for each county was converted to vector abundance as described by Perkins, et al. (*3*) In brief, by assuming that mosquito abundance is distributed as a Poisson random variable, Perkins et al. nicely point out that the expected abundance of mosquitos (λ) in a given location (i) can be calculated from the probability of a mosquito (P_M) at the ith location as $P_{Mi} = 1 - \exp(-\lambda_i)$. Thus, the expected peak abundance of mosquitos available per person per day (λ) can be calculated as $\lambda = -\ln(1 - P_M)$, and λ is thus taken to approximate the maximum mean daily number of mosquitos available per person. Importantly, because the probability of occurrence maps were generated to reflect the extent of the geographic distribution of *Ae. aegypti* and *Ae. albopictus,* as mentioned above, λ is considered to reflect the maximum expected number of mosquitos per person, or the number of mosquitos available per person during peak vector abundance. λ therefore represents an upper bound of *m*, the maximum average number of mosquitos per person per day (calculated separately for each *Aedes* vector) throughout the year.

For each county, simulations were initiated at time of peak vector abundance (see below and Appendix Figure 2) and as simulations progressed, mosquitos available per human changed dynamically. For each simulation, λ was used to initialize the mosquito populations (T_{M_0}) such that: $T_{M_0} = \lambda T_H$, where T_H represents the total human population in the respective county. Simulations were thus initiated during the month of the year in each county when vector abundance was expected to be greatest (see below). Once initialized however, vector populations were modeled explicitly via a series of stochastic ordinary differential equations (ODE) and thus human-mosquito interactions changed dynamically. At peak mosquito abundance (i.e. $\frac{T_M}{T_H} = \lambda$), the rate of mosquito bites per person per day (*m*) was given by $m = \frac{T_M}{T_H}\tau\alpha = \lambda\tau\alpha$, Where τ represents the mean number of daily blood meals taken by each respective *Aedes* vector, and α the proportion of blood meals taken on humans. To account for fluctuating mosquito vector dynamics, which occur over orders of magnitude between summer and winter, the mosquito-human bite rate (m) was constantly updated at every time-step to account for changes in the individual *Aedes* mosquito populations, as well as the number of blood meals taken per day, which is dependent on the duration of the gonotrophic cycle.

Temperature Dependent Functions

For the temperature dependent parameters (described in the following sections), we obtained empiric data reported in the literature across ranges of temperatures, based on relevant papers that studied each individual parameter of interest across the range of relevant temperatures, and fit very simple models (splines, simple linear, simple exponential, etc.) to each set of temperature dependent empiric data. These models were sufficient to capture the parameter of interest's empiric data across temperature gradients for each mosquito aegypti and albopictus where relevant. Once parameterized, for model runs, the simple models were then embedded into simple functions, usually of the form "getZZZperTemp(T = …, mosquito = c("aegypti," "albopictus") where ZZZ refers to, for example, EIP or duration of the gonotrophic cycle at the respective temperature, etc. See code script "2 Create aedes FXNs" for details on individual models describing the empiric parameters over temperature ranges.

Duration of the Gonotrophic Cycle

Duration of the gonotrophic cycle as a function of temperature (g(T)) for each *Aedes* vector species was parameterized from Brady et al. (*9*) In their report, the authors used a large and comprehensive dataset comprised of 54 unique studies of *Aedes aegypti* and *Aedes albopictus* gonotrophic cycles across temperatures to fit a robust enzyme kinetics model to estimate the duration of *Aedes aegypti* and *Aedes albopictus* gonotrophic cycles across temperatures. We used these estimates to parameterize the duration of the gonotrophic cycle at each temperature as simulations moved through time. For average temperatures below those estimated by Brady et al. (<15°C) the expected duration of the gonotrophic cycle greatly exceed the expected lifespan of an adult *Aedes* mosquito by an order of magnitude or more, and thus the duration of the gonotrophic cycle at low temperatures was both not reliably estimated nor important in any of our measured outcomes (data not shown), primarily because mosquito abundance was negligible when average temperatures were low. To allow the model to run, we set gonotrophic cycle duration at these cold temperatures arbitrarily to 100 days. No counties within the U.S. or Puerto Rico had mean high temperatures that exceeded those reported by Brady et al.

Extrinsic Incubation Period (EIP)

Chan et al. (*10*) identified 8 unique studies comprising 146 distinct observations of the extrinsic incubation period to estimate EIP across temperatures. Briefly, the authors used censored time-to-event analyses, and Markov Chain Monte Carlo (MCMC) simulations to compare fits from multiple parametric distributions (Exponential, Weibull, Gamma and Log-normal). Among the fits, Chan et al. found that the log-normal distribution provided the best fit to model EIP as a function of temperature, and we used this fit to parameterize EIP in our model. Although this study was performed only in *Aedes aegypti* mosquitos, Brady et al. (*9*) too addressed EIP and found no important differences in the EIP between the two *Aedes* vectors and, additionally, their estimated EIP durations across temperatures were in strong agreement with Chan et al. We parameterized our model to match Chan's results, primarily because they incorporated a wider temperature range than the Brady 2014 analysis. Of note, owing to a paucity of data on EIP for Zika virus, EIP here is estimated based on the closely related Dengue virus, a limitation of this study. Given the difficulty of measuring EIP, especially in field conditions, coupled with the novelty of the Zika virus epidemic, reliable data to describe the EIP of Zika virus is not yet available and thus measured Dengue virus properties (owing to its close phylogenetic proximity to Zika virus), provides us with our best available estimates for numerous Zika virus properties, a strategy that has been successfully used by others (*3*,*4*). We packaged the log-normal fit provided by Chan et al. into a simple function in R with a single argument (temperature) to be called dynamically, at every time step, for incorporation of continuously updated temperature dependent EIP during simulations.

Aedes lifespan (ν_A)

Using a robust dataset consisting of 410 unique experiments, including 351 laboratory experiments and 59 mark-release-recapture field experiments observing *Aedes aegypti* and *Aedes albopictus* survival, Brady et al. developed survival distributions across a continuous temperature spectrum from 0 to 40°Celcius (*9*). Using these survival distributions for each *Aedes* vector type, we defined temperature specific durations of adult *Aedes* survival as the median duration estimated at each temperature between 0 and 40 C (for temperatures below 0°C, duration of survival was set to zero). The expected lifespan distributions across all temperatures, at 0.1-degree increments can be freely downloaded for both *Aedes aegypti* and *Ae. albopictus* at the following URLs.

Model of adult *Aedes albopictus* (http://dx.doi.org/10.6084/m9.figshare.865035).

Model of adult *Aedes aegypti* (http://dx.doi.org/10.6084/m9.figshare.865034).

Blood Meals per Mosquito per Day (τ)

Blood meals per mosquito per day (τ) was defined as the number of expected feeding events that a mosquito takes per day; calculated as the number of feeds, including interrupted feeding patterns per gonotrophic cycle: two and four for *Ae. Aegypti* and *Ae. Albopictus,* respectively (*9,11–14*), divided by the duration (days) of the gonotrophic cycle.

Clutch Size, Egg, Pupal, and Larval Viability across Temperature

Aedes albopictus

Using captured *Ae. albopictus* mosquitos housed at 8 different temperatures between 5°C and 40°C, Delatte et al. (*15*) monitored the numbers of eggs laid, and their development and viability over 10–19 repetitions per temperature to estimate duration to hatch and egg viability. Delatte et al. also describe larval duration and viability by housing, feeding and monitoring at least ten larvae at each temperature, and repeated this over 8 experiments. The authors then applied various statistical models (see original work (*15*) for complete description) to best approximate temperature dependent functions for durations of egg and larval/pupal development and egg and larval/pupal viability. Additionally, they captured the proportion eggs male versus female at each temperature (*9,15*). We incorporated these parameters – clutch size or number of eggs laid per gonotrophic cycle (used to calculate ε, the mean eggs laid daily per adult female mosquito, as clutch size/duration of the gonotrophic cycle), egg survival (ν_e), egg and larval development times (D_e, D_{LV}), larval survival (ν_{LV}), and proportion eggs female at each temperature into our model by packaging their output into simple functions to enable updated parameters at each time step, based on current temperature in the simulation. Data available for number of eggs and egg survival was limited to temperatures at or above 20°C. However, we found that our simulations were insensitive to differences in number of eggs laid below these temperatures, as gonontrophic cycle duration greatly exceeds predicted lifespan of the adult *Aedes albopictus* mosquito at low temperatures and because larvae generally fail to develop at temperatures below 10 or 15°C. Additionally, because male mosquitos do not contribute to the spread of Zika virus, we took the number of eggs laid to be the fraction expected to be female at

each temperature, which ranged from 41% to 66% (*9*,*15*). The above temperature dependent patterns of *Ae. albopictus* development are in agreement with others (*16*), although we did note that egg viability was shifted up ≈10% in the studies by Dickerson et al. This is most likely because the baseline number of eggs for viability calculations in that study were taken to be only embryonated eggs, while Delatte and others used a denominator consistent with all laid eggs. To incorporate the best-fit model of larval development by temperature (also by Delatte et al.) into our model, we captured the output data from their resulting best-fit curve and packaged it into a simple R function for rapid retrieval and updating of larval development time during simulations. The inverse of the fit represents the duration of larval development, in days, across temperatures.

Aedes Aegypti

Carrington et al. reared captured *Aedes aegypti* mosquitos across temperatures from 16°C to 37°C to determine various *Aedes aegypti* life history traits, including clutch size (number of eggs laid), as well as larval development time and larval survival (ε, D_e, ν_{LV}) (*17*). Among numerous metrics, the authors measured: duration from first blood meal to the first day eggs were observed (gonotrophic cycle duration), number of eggs laid, time to pupation (larval development time) and larval survival. Because proportion of females at each temperature were not available for *Ae. aegypti*, we took proportion of female eggs at each temperature to be the same as the proportions described by Delatte et al. for *Ae. albopictus,* described above (*15*).

For egg viability and egg and larval development rates across temperatures for *Aedes aegypti*, we referred to a comprehensive review and analysis by Eisen et al. (*18*) that analyzed impact of temperature on various developmental and life stage characteristics of *Aedes aegypti* mosquitos. They included all studies (earliest in 1901) that observed at least 100 eggs or 25 larvae and only those that observed hatching rates from eggs kept in water, as would be the case in field conditions.

Courette et al. (*19*) performed a thorough meta-analysis of 49 unique studies, each directed at understanding the relationships between many biotic and abiotic factors likely to contribute to *Aedes aegypti* development. They found temperature to be the primary influencer of *Aedes albopictus and Aedes aegypti* development, and reported larval development time across a range of temperatures from 15 to 37°C. Of note, the larval development rate determined by Courette et al. matches the findings by Eisen et al. (*18*) Similar to methods mentioned above, we

packaged the respective outputs of these works into simple R functions for dynamic temperature dependent retrieval of each parameter during simulations. For durations of egg and larvae/pupae development, we took the inverse of the daily reported rates.

Data

Monthly Temperature Data

Monthly temperature data was retrieved from WorldClim – Global Climate Data (http://worldclim.org), a climate data repository for ecologic modeling and global information systems. The monthly temperature data was retrieved at a resolution of 2.5 arc minutes (grids of ≈4.6 km edges) and mean monthly temperatures for each county or municipality were calculated using border data retrieved from the Global Administrative Areas or GADM database, freely available at http://www.gadm.org, and easily accessible in R using the getData() function of the Raster package (*8*).

County Population Data

Population data per county was collected from the U.S. census bureau at (http://www.factfinder.census.gov/).

Monthly Birth Rates

Throughout the year, the proportion of a population's births fluctuate in a predictable manner across calendar months. To estimate the numbers of children born per month, and thus calculate expected numbers of first, second and third trimester pregnancies per month, the numbers of births per month were collected for each county over a span of 8 years, from 2007 to 2014 from the U.S. Census database. For each county, generalized additive models (GAM's) were fit to the monthly data to model expected proportions of annual births per month per county. For counties with populations fewer than 50,000, stochastic effects masked clear seasonality of human birth rates. Therefore, for those counties with populations below 50,000, the proportion of annual births in each month were pooled within a given state (excluding counties with populations above 50,000) and GAMs were fit to the pooled statewide data. The county-level (for counties ≥50,000) or statewide (counties <50,000) GAM output, indicating the expected proportion of annual births in each calendar month, was then coupled to annual birth numbers for each individual county to calculate monthly county-specific expected pregnancies.

Trimester-Specific Pregnancies and Exposure Calculations

From the monthly birth data for each county, we calculated the number of pregnancies in their first, second or third trimester during each month for each county or municipality. To achieve this, we assumed that within a given month, births were uniformly distributed and that each trimester is 13.33-weeks long, for a 40-week gestation. Therefore, for example, among January births, we assume that all of those pregnancies delivering in January were in their third trimester in January (up until delivery), as well as in December and November, and that half were in their third trimester in October (those with deliveries in the first half of January), and half were in their second trimester in October (those with deliveries in the second half of January). We iterated this routine for each trimester and month for each county to derive numbers of pregnancies in each trimester across all months.

To calculate infections during pregnancy during the simulations, we assumed that pregnancies were among women who remained well-mixed within the population. Given the large numbers of simulations, for efficiency, rather than including numerous compartments for women in each trimester of their pregnancy into our SEIR model, using the assumption of homogenous mixing, we derived the number of fetal exposures per trimester per week for each simulation by drawing from a binomial distribution, with the size equal to the number of first, or second, or third trimester pregnancies in the county, during the week of interest, and with a probability equal to the proportion of the population infected during that week. Therefore, for example, to calculate number of first trimester pregnancies infected during week 36 in a given county, q, in R we coded: rbinom(n = 1, size = n_tri1_wk36_q, prob = prop_Inf_wk36_q), where n_tri1_wk36_q is the number of first trimester pregnancies expected in week 36 for county q, and prop_Inf_wk36_q is the proportion of the county (q) population infected during that week. By drawing from a binomial distribution, we incorporate stochastic effects that influence the number of infections among gravid women, relative to the proportion infected across the population as a whole.

Model

The model is a stochastic SEIR / SEI model with an additional two equations to describe mosquito egg, larval/pupal, and adult development for simulating mosquito populations as functions of temperature throughout the year in each county. The model is defined as follows (Appendix Figure 1 and equations below).

SEIR Model (Human)

$$\frac{dS_H}{dt} = -m\beta_H\left(\frac{I_M}{T_M}\right)S_H$$

$$\frac{dE_H}{dt} = m\beta_H\left(\frac{I_M}{T_M}\right)S_H - E_H\delta^{-1}$$

$$\frac{dI_H}{dt} = E_H\theta\delta^{-1} - \gamma^{-1}$$

$$\frac{dR_H}{dt} = \gamma^{-1}$$

$$\frac{dR_{H2}}{dt} = E_H(1-\theta)\cdot\delta^{-1}$$

Mosquito Development

$$\frac{dEgg}{dt} = \varepsilon T_M - \left(\frac{1-\nu_E}{D_E} + D_E{}^{-1}\right)Egg$$

$$\frac{dLV}{dt} = Egg \cdot D_E{}^{-1} - \left(\frac{1-\nu_{LV}}{D_{LV}} + D_{LV}{}^{-1}\right)LV$$

SEI Model (Mosquitos)

$$\frac{dS_M}{dt} = \frac{LV}{D_{LV}} - \left(\tau\alpha\beta_M\frac{I_H}{T_H} + D_A{}^{-1}\right)S_M$$

$$\frac{dE_M}{dt} = \left(\tau\alpha\beta_M\frac{I_H}{T_H}\right)S_M - E_M(EIP^{-1} + D_A{}^{-1})$$

$$\frac{dI_M}{dt} = \frac{E_M}{EIP} - \frac{I_M}{D_A}$$

Where S_H, E_H, I_H, R_H, R_{Hs} indicate compartments for the susceptible, exposed, infectious, recovered following infectious period, and recovered following non-infectious period, respectively. Additionally, S_M, E_M, I_M, LV and Egg indicate population compartments for susceptible, exposed and infectious mosquitos, as well as larvae and egg populations. Definitions for each of the variables and rate parameters are presented in Appendix Table 1.

Simulations

Simulations were run using an adaptation of the Gillespie Stochastic Simulation Algorithm (SSA), adaptive tau-leaping algorithm on a continuous time scale. Tau-leaping was initially described by Gillespie (*20*). Briefly, tau-leaping procedures achieve efficient implementations of the SSA by relaxing the requirement to model every change (step) in the system – a hugely resource consuming process – by using a Poisson approximation to take "leaps" over fast (stable) steps, while well approximating the stochastic behavior that would be expected over the duration (tau) of the leap. A limitation of tau-leaping however is that the leap size must be pre-specified. When the system is stiff and, by definition predictable, such as during the exponential growth phase of an epidemic, relatively large leaps can be taken because the Poisson approximation is sufficient to approximate the behavior of the system. In fact, under stiff (stable) conditions, with very fast steps and large populations, the tau leaping method limits to the explicit Euler method. However, when the system is nonstiff, and a particular single event can have profound effects on the simulation, i.e., a "critical reaction," such as following introduction of an index case to a community or, more generally, when an extinction of a compartment is possible, the leap sizes must be much smaller. Therefore, if critical reactions are possible, the leap sizes must be reduced, eventually approximating the fundamental Gillespie SSA such that no more than a single critical reaction can take place during a given time-step or leap, also ensuring a population cannot become negative. Because epidemic behavior shifts from nonstiff systems (for example just following introduction of an index case, when extinction of an infectious disease in a community is likely, or when mosquito populations become very low in winter months) to very stiff systems (for example once an epidemic takes off in a susceptible population) declaring a prespecified leap size will either risk missing critical reactions when tau is large but the system is nonstiff, or will be highly inefficient if tau is small but the system is stiff. The adaptive tau-leaping procedure solves this problem by monitoring the stiffness of the system and determines tau by automatically switching between explicit (best for nonstiff systems) and implicit (best for stiff systems) tau selection formulas to determine tau size compatible for the leap conditions at each step. The details of tau selection, including switching between implicit- and explicit-tau selection over the course of a simulation is outside of the scope of this description, and we refer to initial work by Cao, Gillespie and Petzold (*21*).

Simulation Initialization

Initial mosquito abundance per person was given by λ, the maximum mosquito:human ratio described in previous sections and every simulation was initialized at the time of peak vector abundance. For each simulation however, a ‘settling’ period of at least 1 year was incorporated to allow mosquito population dynamics to be driven by their temperature functions, rather than purely by their initialized values. This was important to allow for simulations with index cases introduced at any time of the year, not just at peak vector abundance (initialization). Additionally, this served as a check on the utilization of the high resolution Aedes risk maps. For example, if the risk maps suggested a high density of Aedes aegypti mosquitos in a particular area where temperature constraints should limit mosquito survival, the dynamic temperature dependent *Aedes* life-stage model would serve to temper the *Aedes* population, 1 year later, down from the initial estimated maximum value, to a value dictated more by the temperature and life-stage dynamics. However, we did not detect settings where peak mosquito populations in the year or two after initiation were highly discrepant from the initializing population – further confirming the detailed work by Kraemer et al in their initial development of the mosquito population risk maps.

To determine the start-month for each county’s simulations (i.e., the month of peak *Aedes* abundance when $T_M/T_H = \lambda$ for each county) we ran 36-month simulations for every county and municipality and recorded calendar month when vector abundance was maximized during each year of the simulations. The calendar month recorded to have highest average mosquito population was taken to be the start month of all further simulations for each respective county (this was important to determine because mosquito populations were initialized by their maximum abundance). Nevertheless, sensitivity analysis of the starting month demonstrated no effect of the particular starting month on outcomes, owing to the winter bottleneck during the minimum 1-year ‘settling’ period (these results not shown). This was further confirmed by checking that the month of peak *Aedes* abundance was consistent from year one to year three. Additionally, our findings of month of peak vector abundance are in agreement with results described by Monaghan et al. (*22*,*23*) (Appendix Figure 17) and in a recent report by Grubaugh et al. (*24*)

For each simulation and each vector, Aedes abundance was initialized by λ mosquitos available per person, and the simulation was allowed to progress for at least a full year to allow

mosquito dynamics to be driven by their respective temperature dependent functions, as described above, before a single infected individual (index case) was input into a fully susceptible population at a time specified by the simulation of interest, as described in the main text.

Duration

For each simulation, epidemics were initiated by a single index case and were allowed to progress either until 4 consecutive months passed without any new infections, or for a maximum 60 months.

Design and Analysis

For all of the simulations described in the main text, at least 500 simulations were run for each county or municipality and for each scenario, requiring 1.6 million simulations for a full evaluation of single scenario (for example, a June index case in the setting of 10% vaccination is one scenario). We explored running as many as 1000 simulations per county, however results were generally robust, with minimal added information gained beyond even 200 simulations. Thus, 500 simulations were chosen owing to the already long run times per simulation (3.3 seconds). For the primary results discussed in the manuscript, for a given scenario and county, if 200 simulations passed with no transmission, simulations were halted. In total, over 50 million index cases were simulated for the described results. For each simulation, individual time steps were summarized and stored as daily, weekly or monthly summaries for all events of interest. Summary statistics discussed in the text were calculated from daily summaries (Appendix Table 2).

Computational Environment

All simulations were performed in R installed on Amazon Web Services® elastic cloud (EC2®) instances. Given the large number of full simulations (>50 million index cases and each simulation averaging 3.3 seconds depending on the compute instance), simulations were run in parallel over 160 cores and spread across multiple virtual machine instances. For raw model output and more model information, please see the following data repository: https://github.com/lberylguterman/Zika_ViroImmuno/tree/gh-pages#zika_viroimmuno

Model Comparison

Although the official reporting of confirmed case counts (like all reporting of lab-confirmed cases) under-reports true incidence (i.e., many asymptomatic and symptomatic cases are never laboratory confirmed) the monthly data provided by the Ministry of Health offers an accurate assessment of the monthly fractional abundance of cases, and their relative distribution across the 8 health regions. Of note, this required an assumption that reporting rates were generally consistent across the 8 health regions, which we verified by comparing the overall contribution of positive cases per health region as reported by the Ministry of Health against the fraction of positive blood donor units that originated in each health region, as also reported by Chevalier et al. Indeed, the two matched closely with the exception of the health region of Ponce, which appeared to have a lower overall contribution of blood donor specimens than would be expected based on population size (i.e., Ponce contributed only 7% of all blood donation samples analyzed, which is considerably lower than the Ponce's overall population contribution to the Puerto Rican population, and, given the high Zika virus incidence in Ponce reported by the Ministry of Health, likely represents a reduced relative number of blood donations collected or analyzed from Ponce). Nevertheless, the similarities in the two reports, which are based on primarily orthogonal datasets suggests that the use of the Ministry of Health data to derive fractional abundance per health region is appropriate for development of a validation dataset.

We therefore coupled the monthly fractional abundance to the estimated case counts by Chevalier et al. to derive monthly incidence and case counts between April and August 12th, 2016, which was the duration over which the blood donor screening data was reported.

We used the final monthly incidence estimates from Chevalier et al, assuming a PCR detectable viremic duration of 14 days (which is the detectable duration suggested by U.S. FDA approved Zika virus assays). Note that in their manuscript, Chevalier et al provide estimates across a range of assumed durations of PCR detectable viremia – which is distinct from infectious duration. To derive monthly estimates, we took the product of the cumulative case counts on August 12th for each health region, and the fractional monthly incidence for each health region recorded from the Ministry of Health.

Model output for each county/municipality and month of index case, including county name, state, probability of transmission from the index case, median final incidence and

interquartile range, median exposures during each trimester: 1, 2 or 3. For every county or municipality there is an entry for each month of introduction of the index case, January through December. Medians are calculated across only simulations with at least a single transmission event.

References

1. Ross R. Report on the Prevention of Malaria in Mauritius. Waterlow and Sons (London); 1908.

2. Smith DL, Battle KE, Hay SI, Barker CM, Scott TW, McKenzie FE. Ross, Macdonald, and a theory for the dynamics and control of mosquito-transmitted pathogens. PLoS Pathog. 2012;8:e1002588. PubMed https://doi.org/10.1371/journal.ppat.1002588

3. Perkins TA, Siraj AS, Ruktanonchai CW, Kraemer MUG, Tatem AJ. Model-based projections of Zika virus infections in childbearing women in the Americas. Nat Microbiol. 2016;1:16126. PubMed https://doi.org/10.1038/nmicrobiol.2016.126

4. Bogoch II, Brady OJ, Kraemer MUG, German M, Creatore MI, Kulkarni MA, et al. Anticipating the international spread of Zika virus from Brazil. Lancet. 2016;387:335–6. PubMed https://doi.org/10.1016/S0140-6736(16)00080-5

5. Kraemer MU, Sinka ME, Duda KA, Mylne AQ, Shearer FM, Barker CM, et al. The global distribution of the arbovirus vectors *Aedes aegypti* and *Ae. albopictus*. eLife. 2015;4:e08347. PubMed https://doi.org/10.7554/eLife.08347

6. Kraemer MUG, Sinka ME, Duda KA, Mylne A, Shearer FM, Brady OJ, et al. The global compendium of *Aedes aegypti* and *Ae. albopictus* occurrence. Sci Data. 2015;2:150035. PubMed https://doi.org/10.1038/sdata.2015.35

7. Centers for Disease Control and Prevention. Estimated range of *Aedes albopictus* and *Aedes aegypti* in the United States, 2016 [cited 2016 Apr 1]. http://www.cdc.gov/zika/vector/range.html

8. GADM maps and data [cited 2016 Dec 29]. https://gadm.org

9. Brady OJ, Johansson MA, Guerra CA, Bhatt S, Golding N, Pigott DM, et al. Modelling adult *Aedes aegypti* and *Aedes albopictus* survival at different temperatures in laboratory and field settings. Parasit Vectors. 2013;6:351. PubMed https://doi.org/10.1186/1756-3305-6-351

10. Chan M, Johansson MA. The incubation periods of Dengue viruses. PLoS One. 2012;7:e50972. PubMed https://doi.org/10.1371/journal.pone.0050972

11. Scott TW, Takken W. Feeding strategies of anthropophilic mosquitoes result in increased risk of pathogen transmission. Trends Parasitol. 2012;28:114–21. PubMed https://doi.org/10.1016/j.pt.2012.01.001

12. Scott TW, Clark GG, Lorenz LH, Amerasinghe PH, Reiter P, Edman JD. Detection of multiple blood feeding in *Aedes aegypti* (Diptera: Culicidae) during a single gonotrophic cycle using a histologic technique. J Med Entomol. 1993;30:94–9. PubMed https://doi.org/10.1093/jmedent/30.1.94

13. Gubler DJ. Dengue. In: Epidemiology of arthropod-borne viral disease, Monath TPM, editor. Boca Raton (FL): CRC Press; 1988:223–60.

14. Gubler D, Bhattacharya N. Observations on the reproductive history of *Aedes* (*Stegomyia*) *albopictus* in the laboratory. Mosq News. 1971;31:356–9.

15. Delatte H, Gimonneau G, Triboire A, Fontenille D. Influence of temperature on immature development, survival, longevity, fecundity, and gonotrophic cycles of *Aedes albopictus*, vector of chikungunya and dengue in the Indian Ocean. J Med Entomol. 2009;46:33–41. PubMed https://doi.org/10.1603/033.046.0105

16. Dickerson CZ. The effects of temperature and humidity on the eggs of *Aedes aegypti* (L.) and *Aedes albopictus* (Skuse) in Texas [cited 2016 Dec 29]. https://oaktrust.library.tamu.edu/handle/1969.1/ETD-TAMU-2508

17. Carrington LB, Armijos MV, Lambrechts L, Barker CM, Scott TW. Effects of fluctuating daily temperatures at critical thermal extremes on *Aedes aegypti* life-history traits. PLoS One. 2013;8:e58824. PubMed https://doi.org/10.1371/journal.pone.0058824

18. Eisen L, Monaghan AJ, Lozano-Fuentes S, Steinhoff DF, Hayden MH, Bieringer PE. The impact of temperature on the bionomics of *Aedes* (*Stegomyia*) *aegypti*, with special reference to the cool geographic range margins. J Med Entomol. 2014;51:496–516. PubMed https://doi.org/10.1603/ME13214

19. Couret J, Benedict MQ. A meta-analysis of the factors influencing development rate variation in *Aedes aegypti* (Diptera: Culicidae). BMC Ecol. 2014;14:3. PubMed https://doi.org/10.1186/1472-6785-14-3

20. Gillespie DT. Approximate accelerated stochastic simulation of chemically reacting systems. J Chem Phys. 2001;115:1716–33. https://doi.org/10.1063/1.1378322

21. Cao Y, Gillespie DT, Petzold LR. Adaptive explicit-implicit tau-leaping method with automatic tau selection. J Chem Phys. 2007;126:224101. PubMed https://doi.org/10.1063/1.2745299

22. Monaghan AJ, Morin CW, Steinhoff DF, Wilhelmi O, Hayden M, Quattrochi DA, et al. On the seasonal occurrence and abundance of the Zika virus vector mosquito *Aedes aegypti* in the contiguous United States. PLoS Curr. 2016;8:8. PubMed https://doi.org/10.1371/currents.outbreaks.50dfc7f46798675fc63e7d7da563da76

23. Rund SSC, Martinez ME. Creating a national vector surveillance system: integrated mosquito trap data and digital epidemiology [cited 2016 Dec 29]. https://www.biorxiv.org/content/10.1101/096875v2

24. Grubaugh ND, Ladner JT, Kraemer MUG, Dudas G, Tan AL, Gangavarapu K, et al. Genomic epidemiology reveals multiple introductions of Zika virus into the United States. Nature. 2017;546:401–5. PubMed https://doi.org/10.1038/nature22400

25. Sivan A, Shriram AN, Sunish IP, Vidhya PT. Host-feeding pattern of Aedes aegypti and *Aedes albopictus* (Diptera: Culicidae) in heterogeneous landscapes of South Andaman, Andaman and Nicobar Islands, India. Parasitol Res. 2015;114:3539–46. PubMed https://doi.org/10.1007/s00436-015-4634-5

26. Pinho ST, Ferreira CP, Esteva L, Barreto FR, Morato e Silva VC, Teixeira MG. Modelling the dynamics of dengue real epidemics. Philos Trans A Math Phys Eng Sci. 2010;368:5679–93. PubMed https://doi.org/10.1098/rsta.2010.0278

27. Ferguson NM, Cucunubá ZM, Dorigatti I, Nedjati-Gilani GL, Donnelly CA, Basáñez MG, et al. Countering the Zika epidemic in Latin America. Science. 2016;353:353–4. PubMed https://doi.org/10.1126/science.aag0219

28. World Health Organization. Zika virus disease [cited 2016 Dec 29]. https://www.who.int/news-room/fact-sheets/detail/zika-virus

Appendix Table 1. Model parameter description and estimates

Parameter	Value	Modeled separately per *Aedes* vector
Mosquito infection and development parameters		
m: number of mosquito-human interactions (bites) per day	$f(T_M, T_H, T, \alpha, \tau, \lambda)$ see methods above	Yes
g: gonotrophic cycle (days)	$f(T)$ see methods	Yes
τ: blood meals per day per mosquito	$f(g(T))$; see methods	Yes
α: proportion of blood meals on humans	*Aedes aegypti*: 0.75 (*25*) *Aedes albopictus*: 0.4 (*25*)	Yes
β_M: probability that a bite of an infected human will infect the mosquito	0.75 (*26*,*27*)	No
EIP: extrinsic incubation period	$f(T)$ See methods above	Yes
D_A: adult *Aedes* lifespan	$f(T)$ See methods above	Yes
ε: mean (female) eggs laid per day per mosquito	$f(T)$	Yes

Parameter	Value	Modeled separately per *Aedes* vector
	See methods above	
ν_E: egg viability	*f*(T)<br>See methods above	Yes
D_E: duration to egg hatch	*f*(T)<br>See methods above	Yes
ν_{LV}: larvae/pupae viability	*f*(T)<br>See methods above	Yes
D_{LV}: duration from egg hatch to adult stage (larvae and pupae development duration)	*f*(T)<br>See methods above	Yes
Human infection parameters		
δ: average viral incubation period in humans (days)	6 (*27*,*28*)	No
γ: average duration of infectious period in humans (days)	6 (*27*,*28*)	No
θ: probability that a human who enters the exposed class will become infectious (regardless of symptomatic vs. asymptomatic state)	0.8 (Assumes a fraction of exposed individuals will not become symptomatic nor infectious (distinct from just asymptomatic). Very little information on this parameter, though sensitivity analysis demonstrated results are relatively insensitive.)	No
β_H: probability a bite of an infected mosquito will expose a human	0.75 (*26*,*27*)	No

Appendix Table 2. Model summary statistics

Summary statistic	Description	Figure (numerals) and panels (letters)
Initial transmission probability	The probability that an index case will result in at least a single transmission event. In other words, the fraction of simulations with at least with local transmission event.	1A, 3A, 3B, 3D, 3E, 3G, 3H
Minimal transmission	The idea of defining a county as having 'minimal transmission' is to be highly sensitive to detect places where transmission is possible (even if very unlikely). A county where 'minimal transmission' is achieved is defined as a county that had at least 1 local transmission event occur in at least 0.5% of simulations. Within a given simulation, minimal transmission was considered to be a single local transmission event.	
Proportion infected	Among those simulations where minimal transmission from index cases is achieved, the median proportion of the population infected.	1B
Infections	Among those simulations where minimal transmission from index cases is achieved, the median number infected.	1C, 1E
Incidence per 100,000	Among those simulations where minimal transmission from index cases is achieved, the median number infected per 100,000 population.	1D, 3C, 3F, 3I
Standardized prevalence (per trimester)	Among counties in the southeastern United States and Texas, county-specific prevalence of pregnancy for each respective trimester per month (see Trimester-specific pregnancies and exposure calculations) was standardized for plotting qualitative dynamics on a single scale by scaling to a mean prevalence across months of zero and standard deviation of one.	2A
Standardized incidence	Among those simulations where minimal transmission from index cases is achieved, monthly incidence was standardized for plotting qualitative dynamics on a single scale by scaling to a mean incidence across months of zero and standard deviation of one.	2A
Exposure risk ratios	The relative risk of exposure to Zika virus during the first (Figure 2, panel B) or second trimester (Figure 2, panel C) of pregnancy compared to the risk of Zika virus exposure during the third trimester of pregnancy, using data in Figure 2, panel A. This was calculated by first isolating all simulations per county with at least a minimum number of infections during pregnancy. For each simulation in each county, the total number of infections per trimester was calculated (described in the methods) and divided by the total number of pregnancies to obtain a trimester specific ZKV exposure incidence per simulation. For each county, the median trimester specific ZKV exposure incidence was calculated and the risk ratio per county was taken as the median (across simulations) trimester 1 (Figure 2, panel B) or trimester 2 (Figure 2, panel C) exposure incidence divided by the median trimester 3 exposure incidence. The distribution of these county specific risk ratios are then shown in Figures 2, panel B, C. For this analysis, to detect a seasonal signal from the stochasticity, simulations included were those with at least 25 infections during pregnancy.	2B, 2C
Median number of (simulated) infections during pregnancy	Among those simulations where minimal transmission from index cases is achieved in the southeastern United States, Texas (Figure 2, panel D) and Puerto Rico (Figure 2, panel E, F), the median number infected during pregnancy (see Trimester-specific pregnancies and exposure calculations for more details).	2D, 2E, 2F

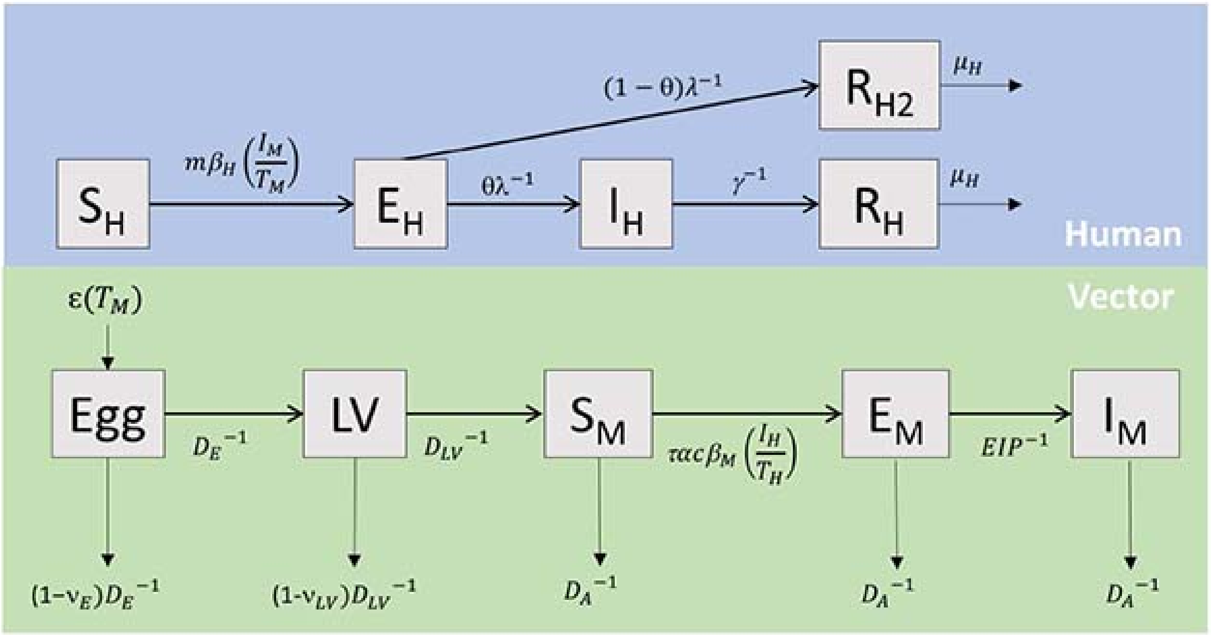

Appendix Figure 1. Compartment diagram of Zika virus transmission model. See methods for model description and definitions.

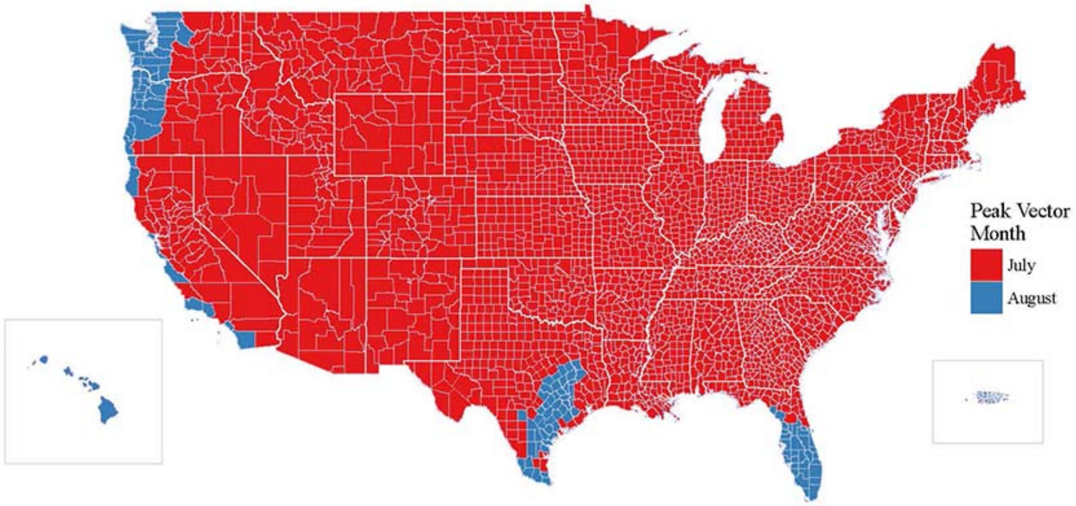

Appendix Figure 2. Month of Peak Vector Abundance. For every county in the United States, we followed Aedes aegypti and Aedes albopictus abundance in our simulations over 3 years following initialization across April - September. Although the actual abundance and fractional abundance over time differed markedly across counties and states, the peak abundance for each county was most often noted in July, with certain counties in Florida, Texas and along the west coast reaching peak abundance in August. Of note, for those counties where vectors were unable to be maintained (i.e. northern states), we initialized simulations as described in the methods for each month between April and September and calculated average mosquito abundance per month for the first year.

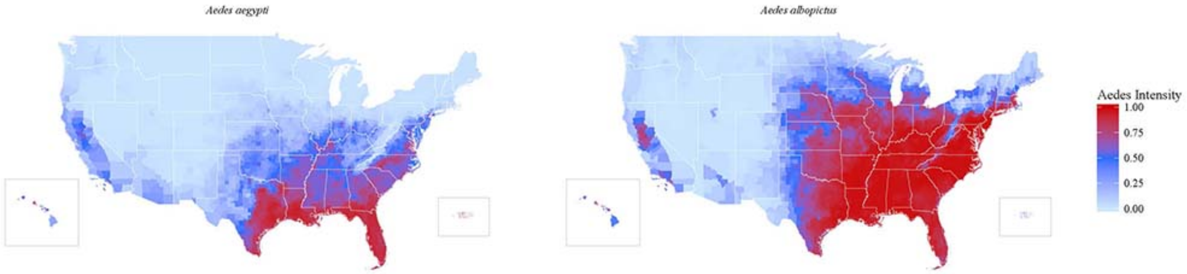

Appendix Figure 3. Predicted intensity of (A) Aedes aegypti and (B) Aedes albopictus mosquitos (developed and described from Kraemer et al. eLife. 2015;4:e08347 and adapted here per county).

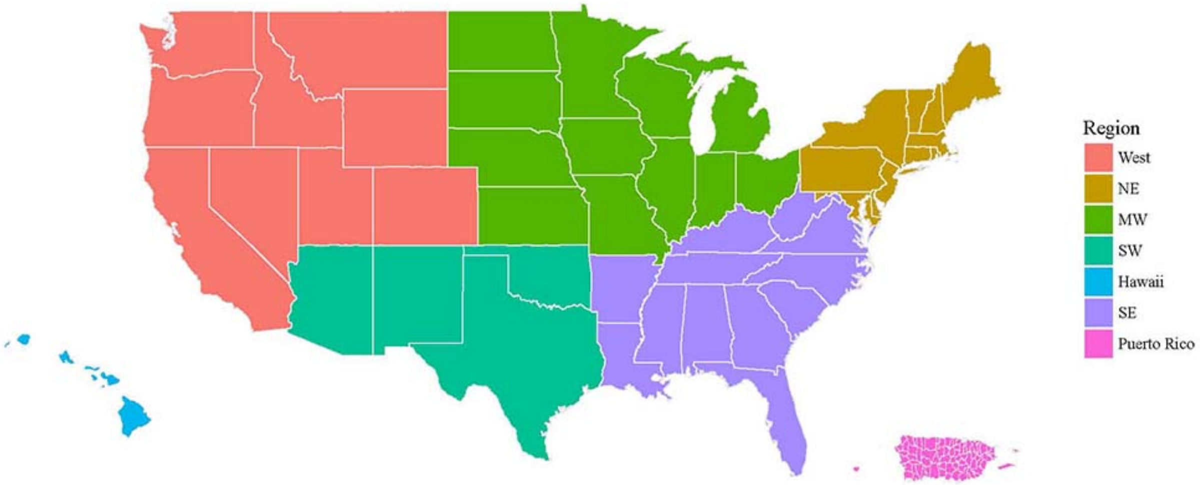

Appendix Figure 4. Regions of the United States. NE = Northeast; MW = Midwest; SW = Southwest; SE = Southeast.

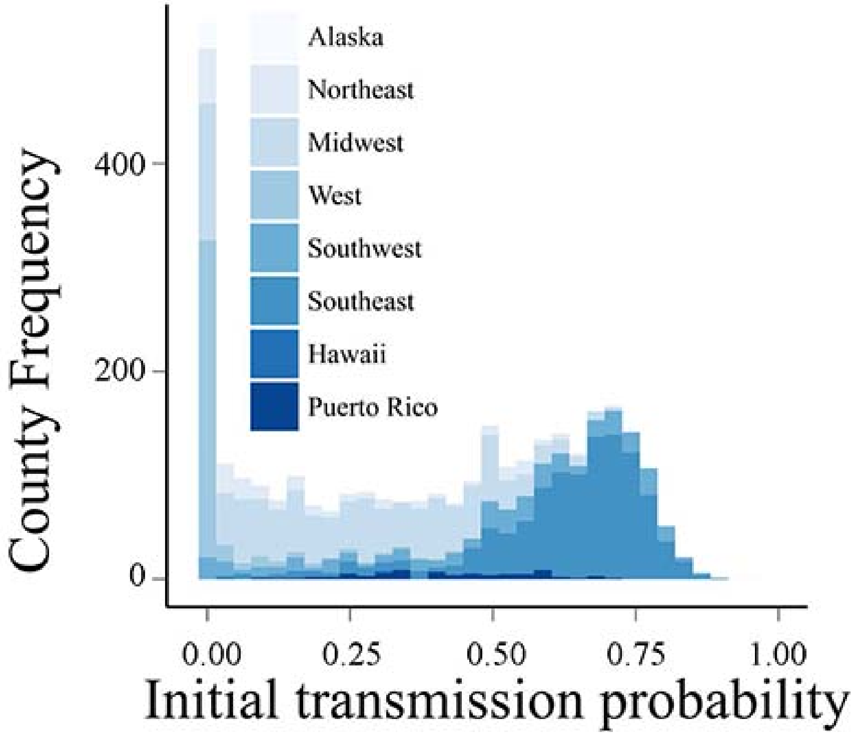

Appendix Figure 5. Probability of initial transmission from an index case introduced during peak vector abundance, calculated as the proportion of simulations with at least a single transmission event, for every county aggregated by region (see Appendix Figure 4 for regions) and displayed as a histogram with county frequency versus probability of transmission from the index case.

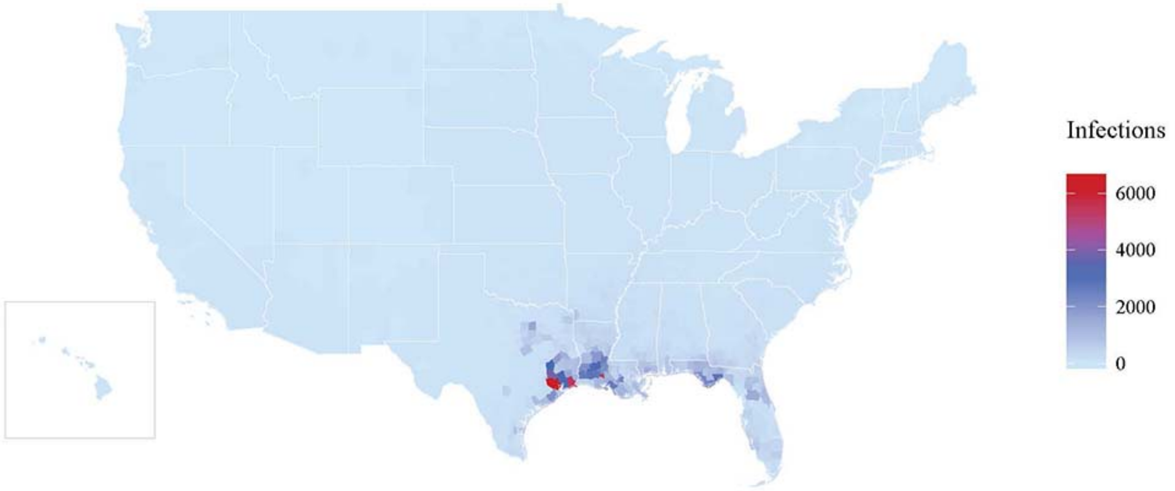

Appendix Figure 6. Total number of infections across the US states. Total number of infections (median across simulations) per county when an index case is imported during peak vector abundance in each respective county.

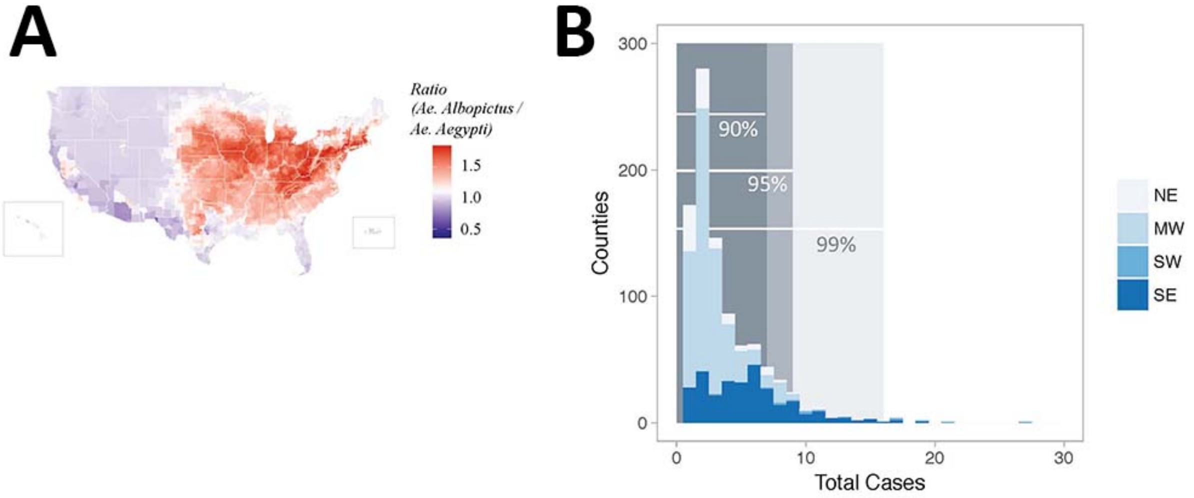

Appendix Figure 7. Total case counts where Aedes albopictus : Aedes aegypti ratio >1.5. (A) Map showing the ratio of relative Aedes albopictus intensity to relative Aedes aegypti intensity (intensities taken from Kraemer et al. (eLife 2015) as described in methods and shown in Appendix Figure 3). (B) Histogram shows that of these counties (those with a ratio >1.5), 90% (dark grey shading), 95% (medium grey), and 99% (light grey shading) have median final case counts of ≤7, ≤9 and ≤16 total cases, respectively. Fewer than 1% of these counties had greater than 20 total median cases.

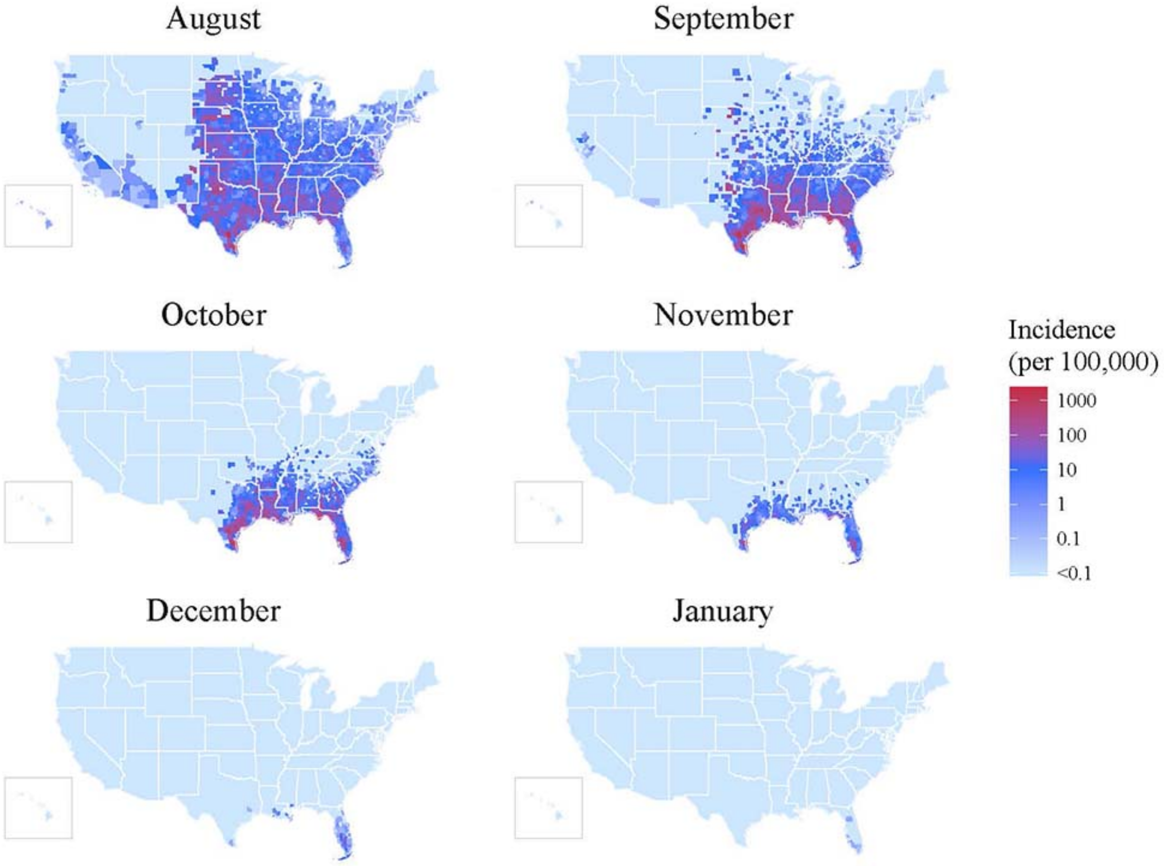

Appendix Figure 8. Monthly incidence of infections in each county following an August index case. Monthly incidence per county when an index case successfully imports into each respective county during the month of August. Incidence reported is the median incidence among simulations with at least a single transmission event.

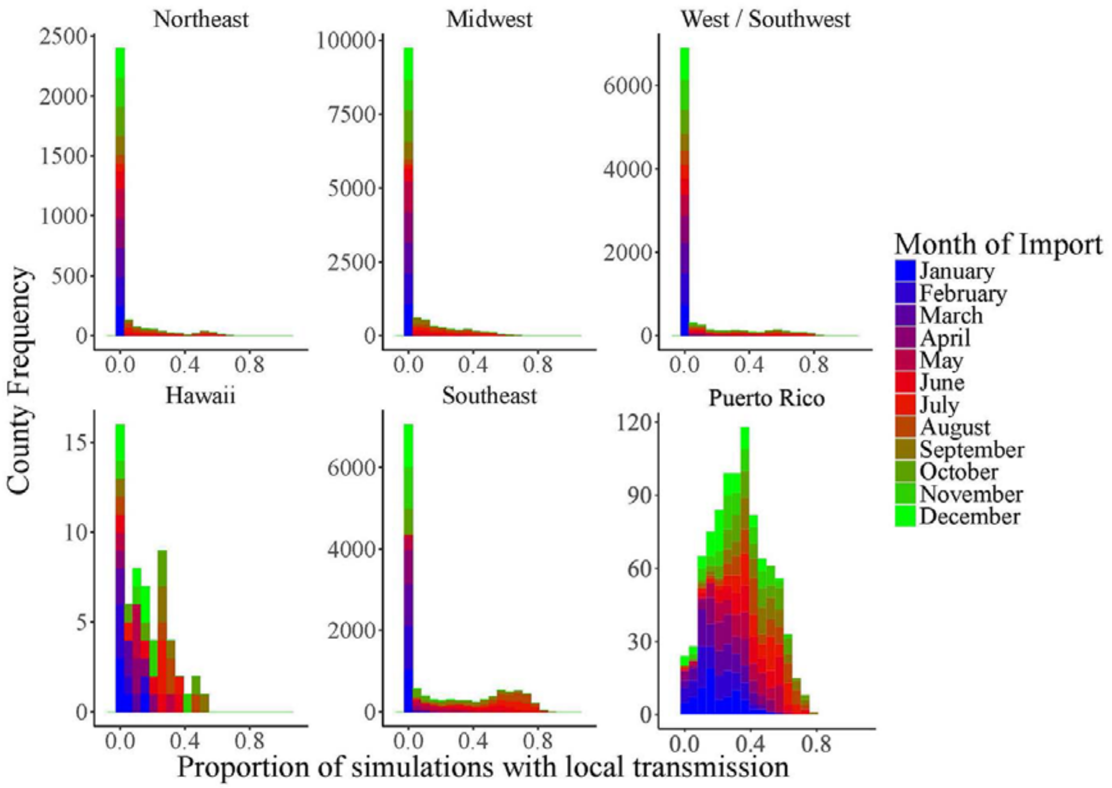

Appendix Figure 9. Probability of transmission from index cases imported across every month. Histogram showing the frequency of counties versus the probability of any transmission from the index case (calculated as the proportion of simulations for which a given county records any transmission) across different months of import. For each of the 3208 counties and municipalities, 200-500 simulations were run for every month of import. January index cases consistently recorded the lowest probability of any transmission, across all regions of the United States and Puerto Rico, while July and August consistently ranked highest for proportion of simulations resulting in any transmission away from the index case. Puerto Rico was the only region that reported initiation of transmission following index cases in every month. Note. Alaska is not reported because it never reported a single transmission event. See Appendix Figure 10 for more detailed results for counties in the southeastern United States.

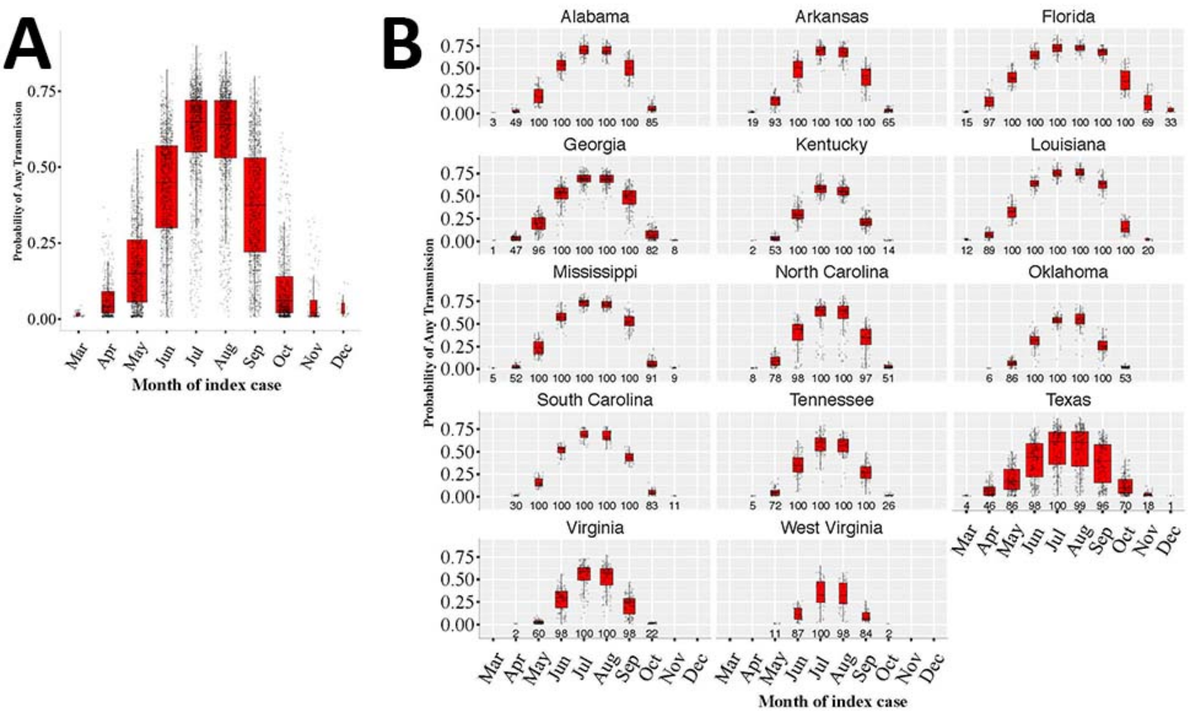

Appendix Figure 10. Probability of initial transmission vs. month of import in the Southeastern United States. Probability of transmission from the index case is plotted for (A) all of the counties in the Southeastern United States and Texas and (B) each of the states individually. Each dot represents a county. Red shading indicates the interquartile range. Counties that fail to achieve at least minimal transmission (i.e. at least a single transmission event in at least 0.05% of simulations) are not plotted, and the width of IQR boxes represents the proportion of total counties included for a given month. Specifically, the width is scaled down from the baseline width in July (the only month with 100% of counties represented) by a factor equal to square root of the number of counties included. In B, the values in each column represent the proportion of counties from the respective state that achieved at least minimal transmission (i.e. the proportion of counties plotted) for a given month of import of the index case.

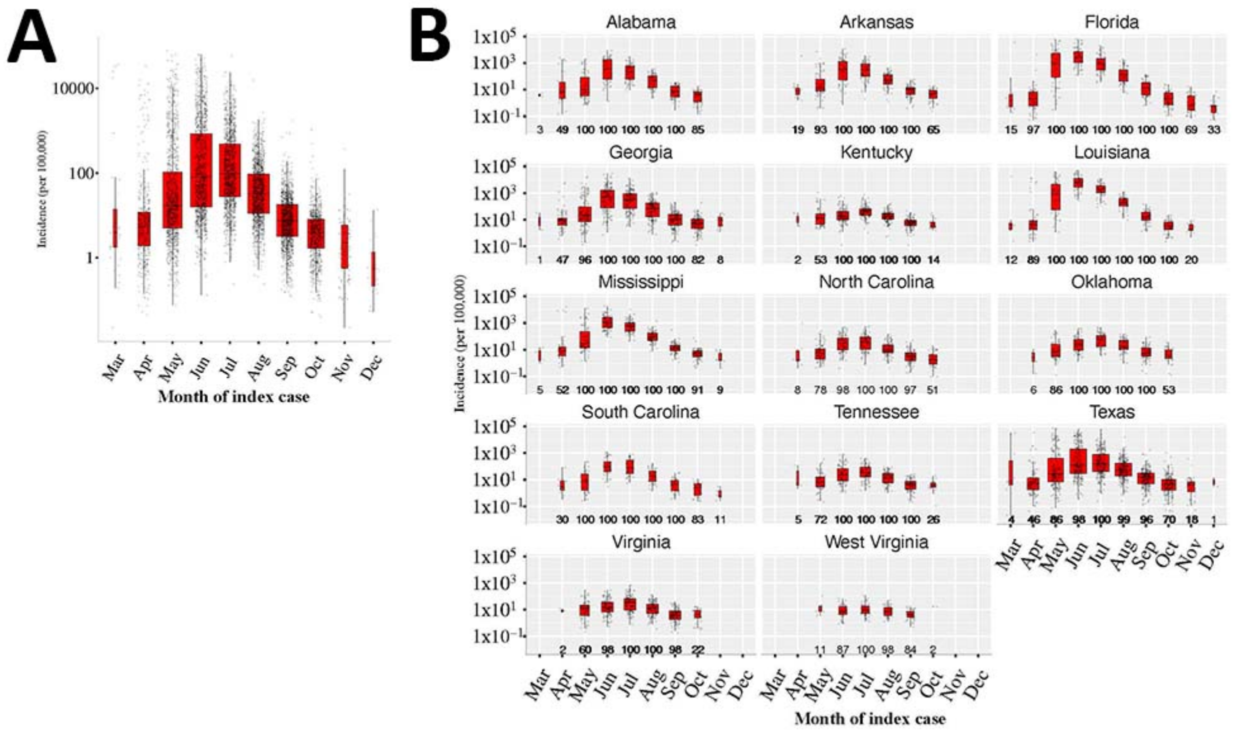

Appendix Figure 11. Incidence vs. month of import in the Southeastern United States. Incidence (median across all simulations with any transmission) of infection is plotted against month of index case for (A) all of the counties in the Southeastern United States and Texas and (B) each of the states individually. Each dot represents a county. Red shading indicates the interquartile range. Counties that fail to achieve at least minimal transmission (i.e. at least a single transmission event in at least 0.05% of simulations) are not plotted, and the width of IQR boxes represents the proportion of total counties included for a given month (as described for Appendix Figure 8). In B, the values in each column represent the proportion of counties from the respective state that achieved at least minimal transmission and are thus plotted for a given month of import. Months with no values and thus no points had no counties that achieved at least minimal transmission.

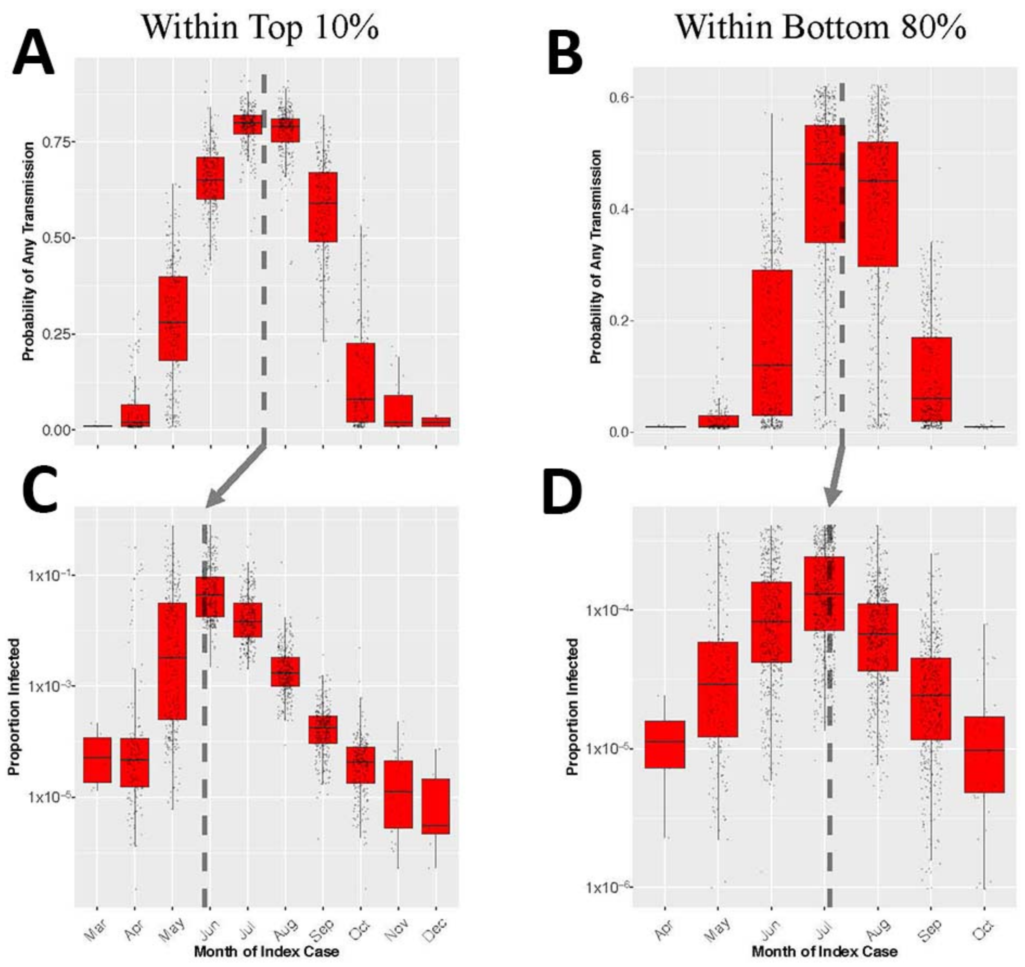

Appendix Figure 12. Probability of any Transmission from the Index case, or proportion infected, versus month of import. (A and B) Probability of at least a single transmission event from the index case (i.e. proportion of simulations with any transmission) versus month of the index case (i.e. month of import) for counties that are within the (A) top 10% or (B) bottom 80% of counties by proportion of simulations with any transmission. For each county, simulations are run separately for each month of import and, for each month of import, the probability of any transmission (i.e. proportion of simulations with at least a single transmission event from the index case) is calculated. For each county, the highest calculated probability (from across the months of import) is then used to bin counties into the top 10% or bottom 80% of counties by probability of achieving any transmission. Once binned, their probabilities of achieving any transmission are then plotted against month of Import, as shown. County-month combinations with zero simulations achieving any transmission are not plotted (as described in Fig S9). (C and D) Proportion of the county population infected, versus month of index case for counties that are within the (C) top 10% or (D) bottom 80% of counties by proportion of population infected. For each county, simulations are run separately for each month of import and, for each month of import, the median proportion of the population infected (among simulations with at least a single transmission event) is calculated. For each

county, the highest median proportion infected (from across the months of import) is then used to bin specific counties into the top 10% or bottom 80% of counties by proportion infected. Once binned, the median proportions infected are plotted for each distinct month of Import, as shown. County-month combinations with no infections are not plotted. The dotted lines represent the mean time of import that achieves the maximum transmission from the index case (A & B) or incidence (C & D). Notable is the difference in the month that maximizes incidence versus the month that maximizes probability of transmission, and that this difference is primarily apparent in the top 10% of counties for each respective metric, but not the bottom 80%, suggesting that among the bottom 80%, transmission chains are not limited by onset of winter, but rather likely die out within a short time-frame, regardless of month of import, and thus overall incidence is driven primarily by mosquito density at time of import.

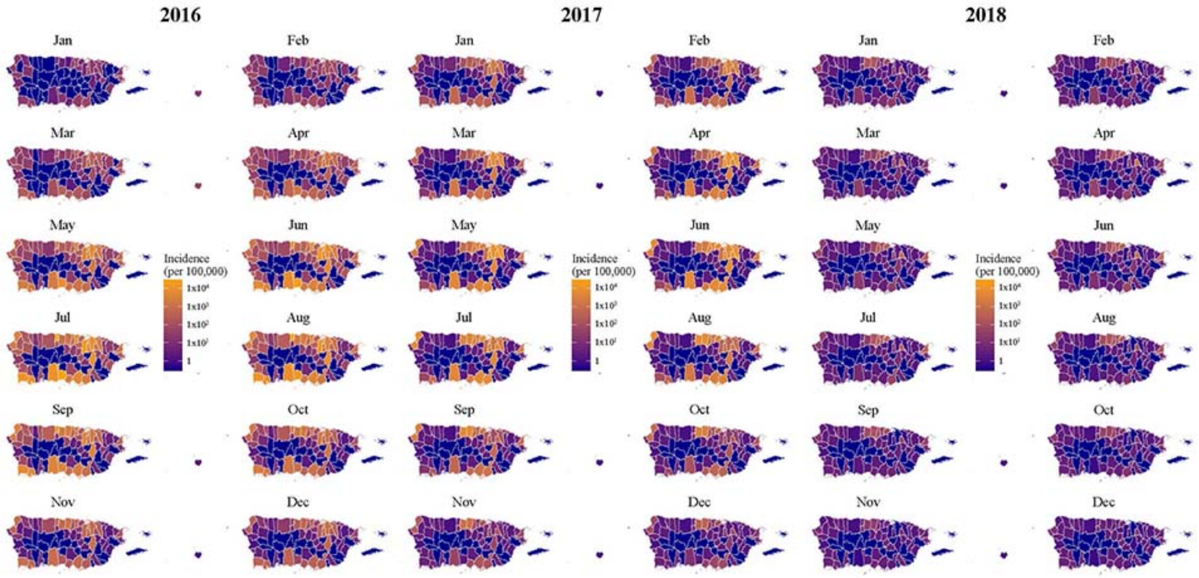

Appendix Figure 13. Monthly incidence of infections throughout Puerto Rico, in the absence of reintroductions. Incidence per month per municipality when index cases were introduced in late 2015 or early 2016 to match timing of initial cases reported in the current epidemic in Puerto Rico. Data shown, and that reported in the text, is absent of reintroductions. Therefore, once an outbreak takes off (cases >10), no reintroductions occur. In the absence of reintroductions here, most municipalities fail to sustain transmission through the winter, suggesting that sustained transmission will be driven by reintroduction events from municipalities where transmission persists uninterrupted, in particular the metropolitan centers of San Juan, Bayamón, Caguas and Ponce. Beyond the first year, these estimates likely underestimate the true state-wide incidence because reintroduction events are expected to be common due to human movement.

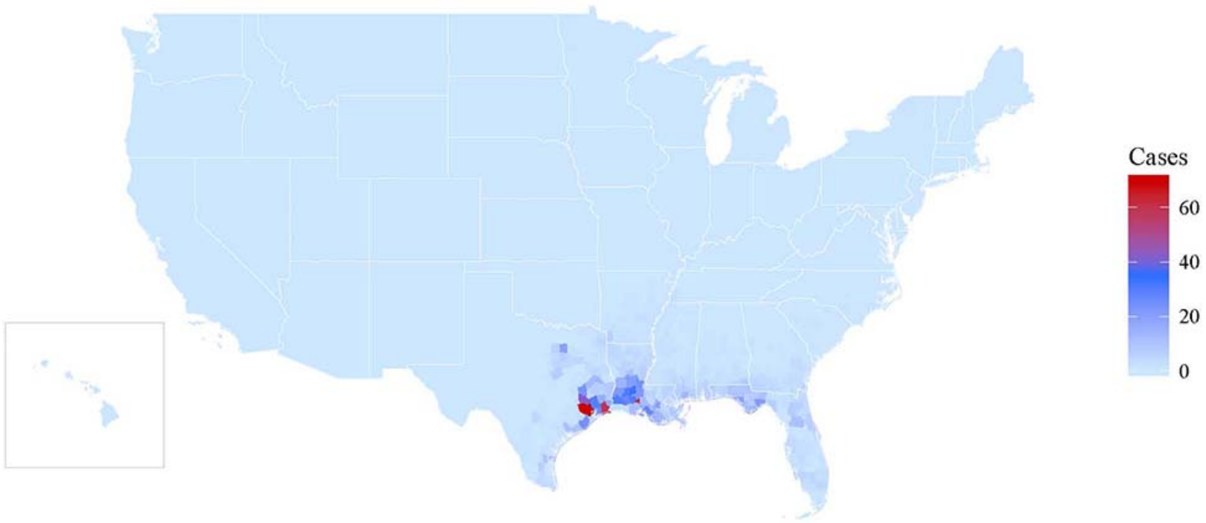

Appendix Figure 14. Zika infections during pregnancy. Total (median) number of zika infections among pregnant women following index cases imported when vectors are at peak abundance.

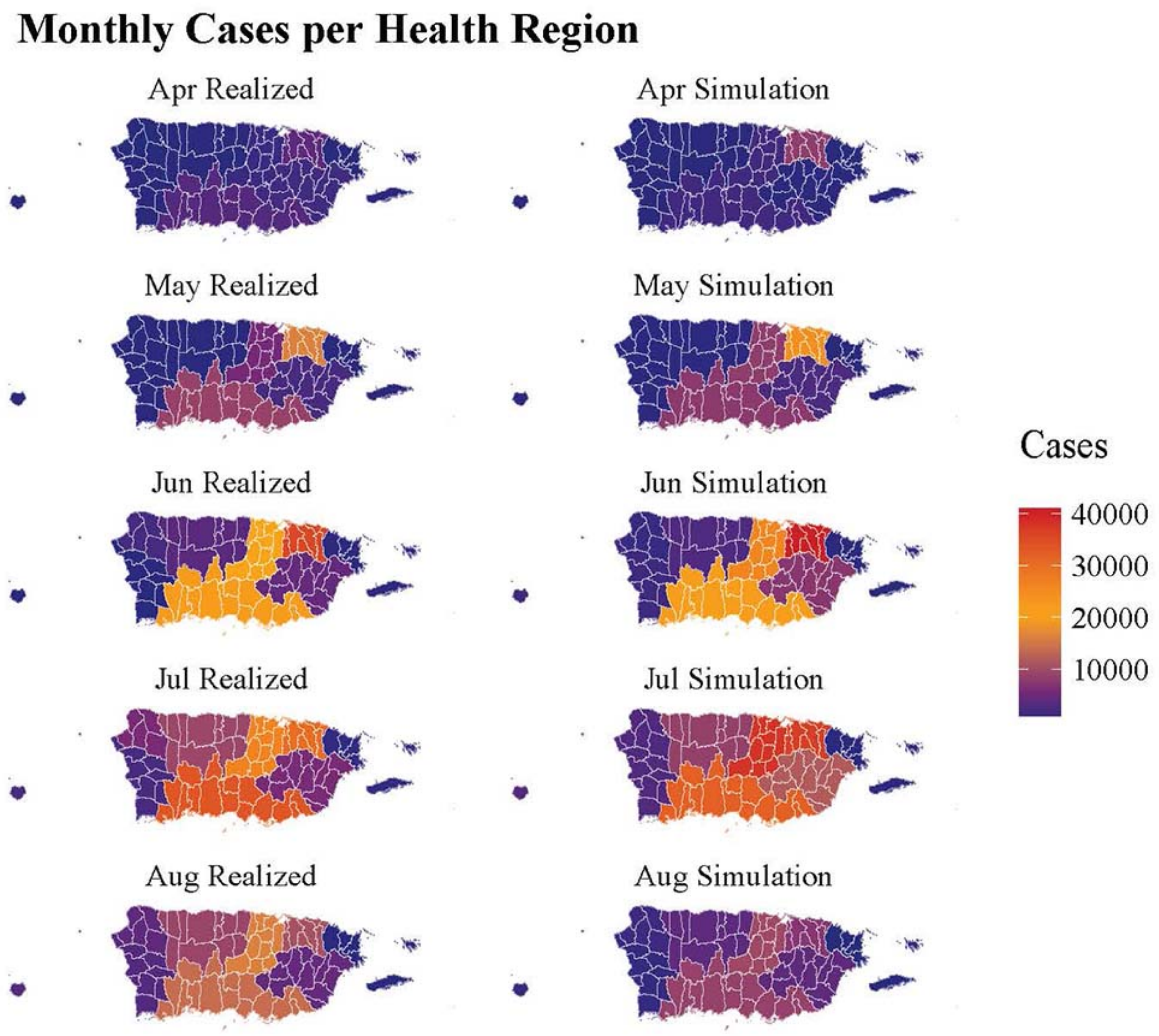

Appendix Figure 15. Monthly numbers of infections per health region realized and simulated. In the left column. Monthly numbers of Zika exposures per health region were estimated from a combination of blood donor screening data estimates (available from April through August 12th, 2016) and surveillance data freely available from the Puerto Rican Ministry of Health (as described in methods). In the right column, simulated monthly numbers of exposures were tallied over the same duration (April – August 12th) from the median simulations for each municipality. As described in the methods and main text, simulations in each municipality were initiated (i.e. index case introduced) to align temporally with initial reported cases in each health municipality, reported and freely available by the Puerto Rican Ministry of Health. Although in our analyses reintroduction events were not generally introduced into any counties, for these particular validation studies, if a particular municipality in Puerto Rico failed to elicit at least 10 infections before the outbreak died-off, a second, or at a maximum a third reintroduction event (i.e. index case) was allowed.

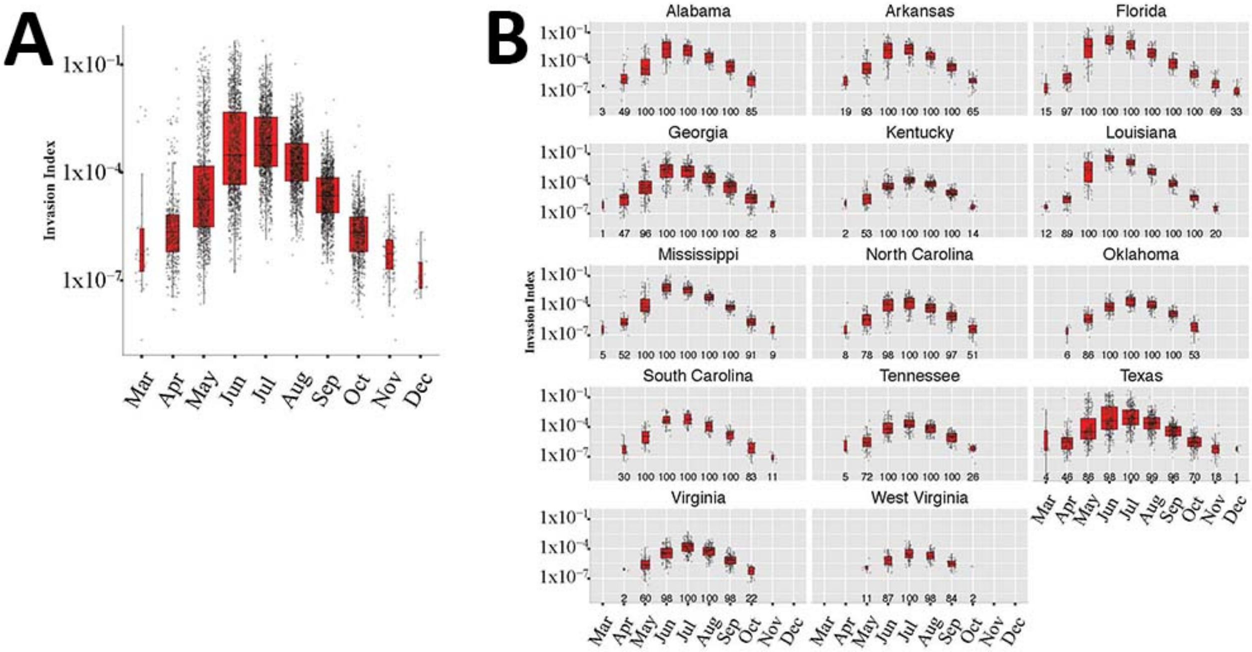

Appendix Figure 16. Invasion Index vs. month of import in the Southeastern United States and Texas. The overall Invasion Index is a composite derived, for each county and each scenario (i.e. month of index case) from the product of the probability that any transmission will occur (Appendix Figure 10) and the median incidence (as the proportion of the population infected; Appendix Figure 11) when transmission does occur. It is therefore neither a measure of the probability of any transmission from the index case, nor the expected incidence, but rather represents an attempt to capture these two important metrics in a single metric to describe an overall or absolute risk for a given location. Whereas, on average, May imports led to the largest epidemics (Appendix Figure 10) and July imports were often most likely to result in transmission (Appendix Figure 9), June imports maximize the overall 'invasion index'. Each dot represents a county. Red shading indicates the interquartile range. Counties that fail to achieve at least minimal transmission (i.e. at least a single transmission event in at least 0.05% of simulations) are not plotted, and the width of IQR boxes represents the proportion of total counties included for a given month (as described for Appendix Figure 9)

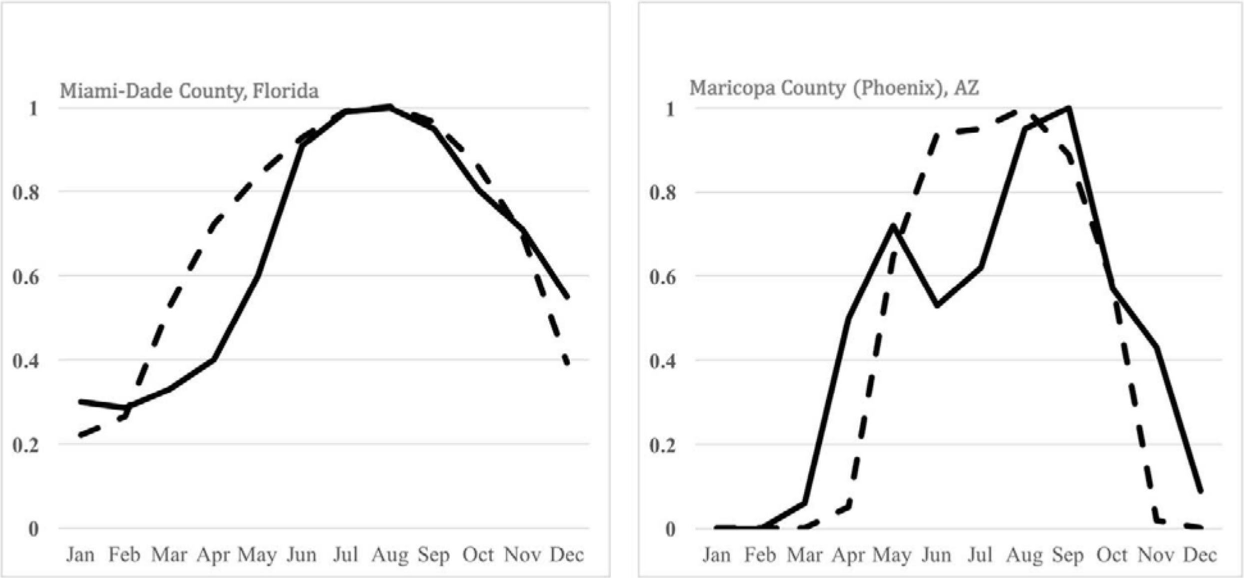

Appendix Figure 17. Example mosquito population dynamics for Miami-Dade County. In agreement with *Aedes aegypti* populations reported by Grubaugh et al (Nature 2017), *Aedes aegypti* increase above their winter baseline in March/April, peak in June – August and finally retreat to low levels again in November/December.
